# Role of Gate-16 and Gabarap in Prevention of Caspase-11-Dependent Excess Inflammation and Lethal Endotoxic Shock

**DOI:** 10.3389/fimmu.2020.561948

**Published:** 2020-09-15

**Authors:** Naoya Sakaguchi, Miwa Sasai, Hironori Bando, Youngae Lee, Ariel Pradipta, Ji Su Ma, Masahiro Yamamoto

**Affiliations:** ^1^Department of Immunoparasitology, Research Institute for Microbial Diseases, Osaka University, Osaka, Japan; ^2^Laboratory of Immunoparasitology, WPI Immunology Frontier Research Center, Osaka University, Osaka, Japan

**Keywords:** GBP2, Gate-16, caspase-11, non-canonical inflammasome, sepsis

## Abstract

Sepsis is a life-threating multi-organ disease induced by host innate immunity to pathogen-derived endotoxins including lipopolysaccharide (LPS). Direct sensing of LPS by caspase-11 activates inflammasomes and causes lethal sepsis in mice. Inhibition of caspase-11 inflammasomes is important for the prevention of LPS-induced septic shock; however, whether a caspase-11 inflammasome-specific suppressive mechanism exists is unclear. Here we show that deficiency of GABARAP autophagy-related proteins results in over-activation of caspase-11 inflammasomes but not of canonical inflammasomes. *Gate-16*^−/−^*Gabarap*^−/−^ macrophages exhibited elevated guanylate binding protein 2 (GBP2)-dependent caspase-11 activation and inflammatory responses. Deficiency of GABARAPs resulted in formation of GBP2-containing aggregates that promote IL-1β production. High mortality after low dose LPS challenge in *Gate-16*^−/−^*Gabarap*^−/−^ mice primed with poly(I:C) or polymicrobial sepsis was ameliorated by compound GBP2 deficiency. These results reveal a critical function of Gate-16 and Gabarap to suppress GBP2-dependent caspase-11-induced inflammation and septic shock.

## Introduction

Sepsis is defined as a life-threatening multi-organ dysfunction syndrome caused by the excessive induction of host innate immunity against microbial infection (1). Even in developed countries, mortality in patients with severe sepsis is 20–50% (2). Various microbes contain endotoxins that have critical roles in the induction of sepsis (3). Lipopolysaccharide (LPS), a cell-wall component of Gram-negative bacteria, is a major endotoxin that strongly stimulates host innate immunity (4). Extracellular LPS is recognized by cell surface receptor complexes containing Toll-like receptor 4 (TLR4) together with CD14, LPS binding protein (LBP), and Myeloid Differentiation factor 2 (MD-2). LBP binds to LPS and then transfers this complex to CD14 to promote the formation of a complex containing LPS and MD-2/TLR4 (5). Activation of TLR4 signaling cascades mediates the production of proinflammatory cytokines such as TNF-α, IL-6, and IL-12, and precursor (preform) proteins of IL-1β and IL-18 (proIL-1β and proIL-18), which are critical for tissue damage and high fever during sepsis (6).

Although extracellular LPS is detected by TLR4, intracellular LPS is sensed by inflammatory caspases (caspase-11 in mice and caspase-4/5 in humans), culminating in activation of the NOD-like receptor protein 3 (NLRP3)-dependent inflammasome (7–9). The NLRP3 inflammasome is a multi-protein complex that contains apoptosis-associated speck-like protein containing a CARD (ASC) and caspase-1 in addition to NLRP3 (10–13). Activation of the NLRP3 inflammasome mediates inflammatory cell death termed pyroptosis and the maturation of proIL-1β and proIL-18 to IL-1β and IL-18, respectively (14). Other NLRP3 specific ligands such as reactive oxygen species (ROS), ATP, pore forming toxins (nigericin) and extracellular crystals, activate canonical NLRP3 inflammasomes independently of caspase-4/5 or caspase-11 (13, 15–17). In contrast, the LPS-mediated activation of NLRP3 inflammasomes critically requires caspase-11 upstream of NLRP3, termed the caspase-11 inflammasome or non-canonical inflammasome (7). The physiological importance of caspase-11 inflammasomes was determined in mice pre-treated with poly(I:C), which developed TLR4-independent but caspase-11-dependent sepsis (18, 19). Thus, caspase-11-dependent pathways are important for LPS-induced sepsis. The negative regulation of NLRP3 inflammasome activation plays a pivotal role in the prevention of excessive inflammation that is detrimental to the host. Inhibitory mechanisms for the canonical NLRP3 inflammasome pathway at multiple steps have been extensively studied (20–30). Regarding a negative regulatory mechanism specific for the caspase-11 inflammasome pathway, Nedd4 and SERPINB1 have been recently reported as inhibitors of non-canonical inflammasomes (31, 32).

Infection with Gram-negative bacteria such as *Salmonella, Citrobacter, Chlamydia*, and *Escherichia* into host innate immune cells activates the caspase-11 inflammasome (7). During the course of an infection, Gram-negative bacteria actively or passively invade into host cells and eventually form pathogen-containing vacuoles (PCVs) (33). Caspase-11 recognition of LPS from Gram-negative bacteria inside PCVs was dramatically enhanced through lysis of the PCVs by interferon-inducible GTPases such as guanylate binding proteins (GBPs), which normally function as interferon-inducible cell-autonomous effectors against various PCV-forming intracellular pathogens such as *Toxoplasma* and Gram-negative bacteria (33). Upon infection by vacuolar pathogens, GBPs are recruited onto the PCV membranes to destroy the structure (34, 35). During Gram-negative bacterial infection, the accumulation of GBPs on PCVs is thought to promote the lysis of PCVs or destroy the bacterial cell wall, resulting in the exposure of LPS to the cytosol and its recognition by caspase-11 (33, 36). Thus, GBP is involved in the activation of caspase-11 inflammasomes and acts as a hub for innate immunity and anti-microbial cell-autonomous immunity.

The critical step for GBP-dependent anti-microbial cell-autonomous immunity is the recruitment of GBP on PCVs, which is regulated by the enzymatic action of autophagy related (Atg) proteins such as Atg3, Atg5, Atg7, and Atg16L1 (37–39). Atg proteins are essential regulators of autophagy, a fundamental eukaryotic biological pathway for the degradation of cellular components (40, 41). The Atg3/Atg5/Atg7/Atg16L1 complex is required for the lipidation of Atg8 family proteins consisting of LC3 (Lc3a and Lc3b in mice) and GABARAP [Gabarap, Gabarapl1 and Gate-16 (Gabarapl2)] subfamilies during autophagy (40). Atg8 family members in addition to Atg3/Atg5/Atg7/Atg16L1 act as positive regulators for GBP-dependent anti-microbial cell-autonomous immunity (42, 43). However, role of the autophagy proteins in GBP-dependent innate immunity such as the caspase-11 inflammasome is unknown.

Here we demonstrate that GABARAP autophagy proteins negatively regulate GBP2-dependent caspase-11 inflammasome activation to prevent sepsis. Depletion of the GABARAP subfamily, but not the LC3 subfamily, in macrophages resulted in enhanced IL-1β production and pyroptosis in response to LPS transfection, OMV treatment and Gram-negative bacterial infection. In contrast, the GBP2-independent LPS introduction–induced activation of caspase-11 inflammasome as well as the ATP-mediated activation of the canonical NLRP3 inflammasome were normal in *Gate-16*^−/−^*Gabarap*^−/−^ cells. Deficiency of Gate-16 and Gabarap resulted in formation of GBP2 aggregates also containing LPS. Moreover, *Gate-16*^−/−^*Gabarap*^−/−^ mice exhibited high susceptibility to LPS-induced and cecal ligation puncture (CLP)-induced septic shock, which was ameliorated by GBP2 deficiency. Taken together, these data demonstrate that GABARAP autophagy proteins specifically limit GBP2-dependent caspase-11 inflammasome activation and sepsis.

## Results

### Elevated IL-1β Production and Pyroptosis in LPS-Transfected *Gate-16*^−/−^*Gabarap*^−/−^ Macrophages

**Figure 1:**
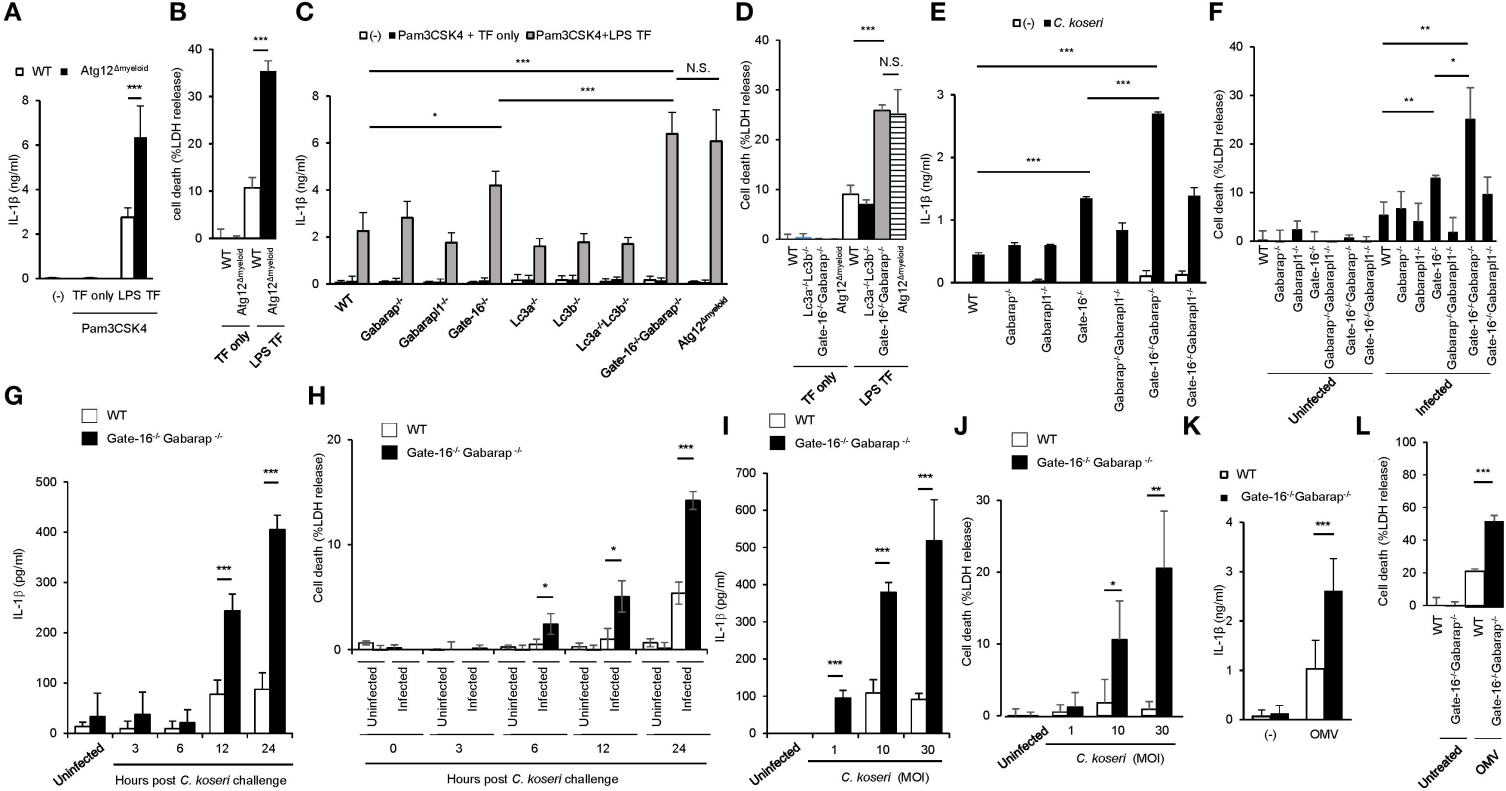
Elevated caspase-11-mediated release of IL-β and pyroptosis in *Gate-16*^−/−^*Gabarap*^−/−^ macrophages. **(A,B)** Release of IL-1β **(A)** or LDH **(B)** from wild-type (WT) and *Atg12*^fl/fl^ BMDMs primed with Pam3CSK4 followed by LPS transfection for 16 h. **(C,D)** Release of IL-1β **(C)** or LDH **(D)** from wild-type (WT), *Atg12*^fl/fl^, *Lc3a*^−/−^*, Lc3b*^−/−^, *Gate-16*^−/−^*, Gabarap*^−/−^*, Gabarapl1*^−/−^*, Lc3a*^−/−^*Lc3b*^−/−^, and *Gate-16*^−/−^*Gabarap*^−/−^ BMDMs primed with Pam3CSK4 followed by LPS transfection for 16 h. **(E,F)** Release of IL-1β **(E)** or LDH **(F)** from wild-type (WT), *Gate-16*^−/−^*, Gabarap*^−/−^*, Gabarapl1*^−/−^*, Gate-16*^−/−^*Gabarap*^−/−^*, Gate-16*^−/−^*Gabarapl1*^−/−^, and *Gabarap*^−/−^*Gabarapl1*^−/−^ BMDMs pre-treated with IFN-γ for 24 h and subsequently infected with *C. koseri* for 24 h. **(G–J)** Release of IL-1β **(G,I)** or LDH **(H,J)** from wild-type (WT) and *Gate-16*^−/−^*Gabarap*^−/−^ BMDMs pre-treated with IFN-γ for 24 h and followed by *C. koseri* infection for indicated hours at MOI **(G,H)** or for 24 h at indicated MOI **(I,J)**. **(K,L)** Release of IL-1β **(K)** or LDH **(L)** from wild-type (WT) and *Gate-16*^−/−^*Gabarap*^−/−^BMDMs stimulated with OMVs for 16 h. The data are combined data of more than three independent experiments. ^*^P < 0.05, ^**^P < 0.01, ^***^P < 0.001, and N.S., not significant, two-tailed *t*-test.

The lysis of bacteria-containing vacuoles by GBPs is important for the activation of caspase-11 inflammasomes (33, 36). Furthermore, we recently showed that some essential autophagy proteins play a critical role in GBP-dependent anti-microbial cell-autonomous immunity (42, 43). To analyze the roles of autophagy proteins in caspase-11-mediated immune responses, we measured LPS transfection-induced IL-1β production and pyroptosis in macrophages from Lysozyme M-Cre Atg12^fl/fl^ mice (Atg12^Δmyeloid^ mice) (Figures 1A,B). Notably, Atg12^Δmyeloid^ macrophages showed elevated IL-1β production and significantly increased LDH release compared with wild-type control cells (Figures 1A,B). Because Atg12^Δmyeloid^ cells (and other cell types below) are particularly sensitive to LPS transfection, we modified the LPS transfection protocol slightly to avoid saturation, resulting in somewhat lower death rate than typically reported using standard protocol (33). Atg12 together with Atg5 and Atg16L1 are critical for the processing of Atg8 family proteins consisting of Lc3a, Lc3b, Gabarap, Gabarapl1, and Gate-16 in mice (35). Therefore, we examined which Atg8 proteins were responsible for the enhanced IL-1β production and pyroptosis in Atg12^Δmyeloid^ macrophages (Figures 1C,D). Among macrophages lacking each of the Atg8 proteins, *Gate-16*^−/−^ cells showed slightly but significantly upregulated IL-1β production, whereas the other deficient cells did not (Figure 1C). Furthermore, *Gate-16*^−/−^*Gabarap*^−/−^ macrophages exhibited much higher levels of IL-1β production and LDH release in response to LPS transfection with a magnitude comparable to Atg12^Δmyeloid^ cells (Figures 1C,D). In contrast, macrophages lacking Lc3a and Lc3b (*Lc3a*^−/−^*Lc3b*^−/−^ macrophages) had similar responses to wild-type cells after LPS transfection (Figures 1C,D), indicating that a lack of GABARAP subfamily proteins such as Gate-16 and Gabarap leads to elevated non-canonical inflammasome responses to LPS transfection. Similar to LPS transfection, *Gate-16*^−/−^*Gabarap*^−/−^ macrophages among cells lacking single or compound deficiency of GABARAP subfamily members were the most hyper-responsive to *Citrobacter koseri*, a Gram-negative bacterium whose infection causes caspase-11 inflammasome activation (33) (Figures 1E,F). Furthermore, excess IL-1β production and cell death in *C. koseri*-infected *Gate-16*^−/−^*Gabarap*^−/−^ macrophages were observed in manners dependent on bacterial dose and infection time (Figures 1G–J). Outer membrane vesicles (OMVs) derived from Gram-negative bacteria contain LPS that stimulates the caspase-11 inflammasome (44). OMV-induced production of IL-1β and induction of cell death were also enhanced in *Gate-16*^−/−^*Gabarap*^−/−^ macrophages (Figures 1K,L). Thus, caspase-11 inflammasome-mediated immune responses were increased by a deficiency of GABARAP subfamily proteins.

### Normal Activation of Canonical NLRP3, Aim2 and NLRC4 Inflammasomes in *Gate-16*^−/−^*Gabarap*^−/−^ Macrophages

To assess whether GABARAPs are specifically involved in caspase-11 inflammasomes, we examined the canonical NLRP3 inflammasome activation in *Gate-16*^−/−^*Gabarap*^−/−^ macrophages (Supplementary Figures 1A–E). ATP-induced IL-1β production and cell death in *Gate-16*^−/−^*Gabarap*^−/−^ cells were similar to wild-type cells (Supplementary Figures 1A,B). Regarding caspase-1 activation, *Gate-16*^−/−^*Gabarap*^−/−^ cells and wild-type cells showed normally induced caspase-1 cleavage in response to ATP stimulation (Supplementary Figure 1C), consistent with normal IL-1β production and pyroptosis (Supplementary Figures 1A,B). In contrast, *Lc3a*^−/−^*Lc3b*^−/−^ macrophages and Atg12-deficient cells were hyper-responsive to ATP (Supplementary Figures 1A,B). ATP-induced mitochondrial damage was previously shown to be elevated in *Lc3b*^−/−^ macrophages due to defective autophagic clearance of damaged mitochondria in LC3-deficient cells with increased canonical inflammasome activation (45). We also found that ATP-stimulated *Lc3a*^−/−^*Lc3b*^−/−^ macrophages and Atg12-deficient cells contained more damaged mitochondria than wild-type cells (Supplementary Figures 1D,E). In sharp contrast, ATP-stimulated *Gate-16*^−/−^*Gabarap*^−/−^ cells did not possess such damaged mitochondria (Supplementary Figures 1D,E). Taken together, these data suggest that, although Atg12-deficiency leads to enhanced activation of both canonical and caspase-11 inflammasomes, Gate-16/Gabarap deficiency selectively over-activates caspase-11 inflammasome. Moreover, poly-dAdT or flagellin transfection-induced IL-1β production in Pam_3_CSK_4_-primed *Gate-16*^−/−^*Gabarap*^−/−^ cells displayed normal IL-1β production (Supplementary Figures 1F,G), suggesting normal Aim2 and NLRC4 inflammasome activation in *Gate-16*^−/−^*Gabarap*^−/−^ cells (11). Moreover, inflammasome-independent IL-6 production in *Gate-16*^−/−^*Gabarap*^−/−^ macrophages in response to Pam3CSK4 stimulation and LPS transfection was comparable to that in wild-type cells (Supplementary Figure 1H). Thus, the GABARAP subfamily proteins are not involved in regulation of Aim2 or NLRC4 inflammasomes, and in inflammasome-independent cytokine production.

### Enhanced Caspase-11-Dependent Signaling in *Gate-16*^−/−^*Gabarap*^−/−^ Macrophages

**Figure 2:**
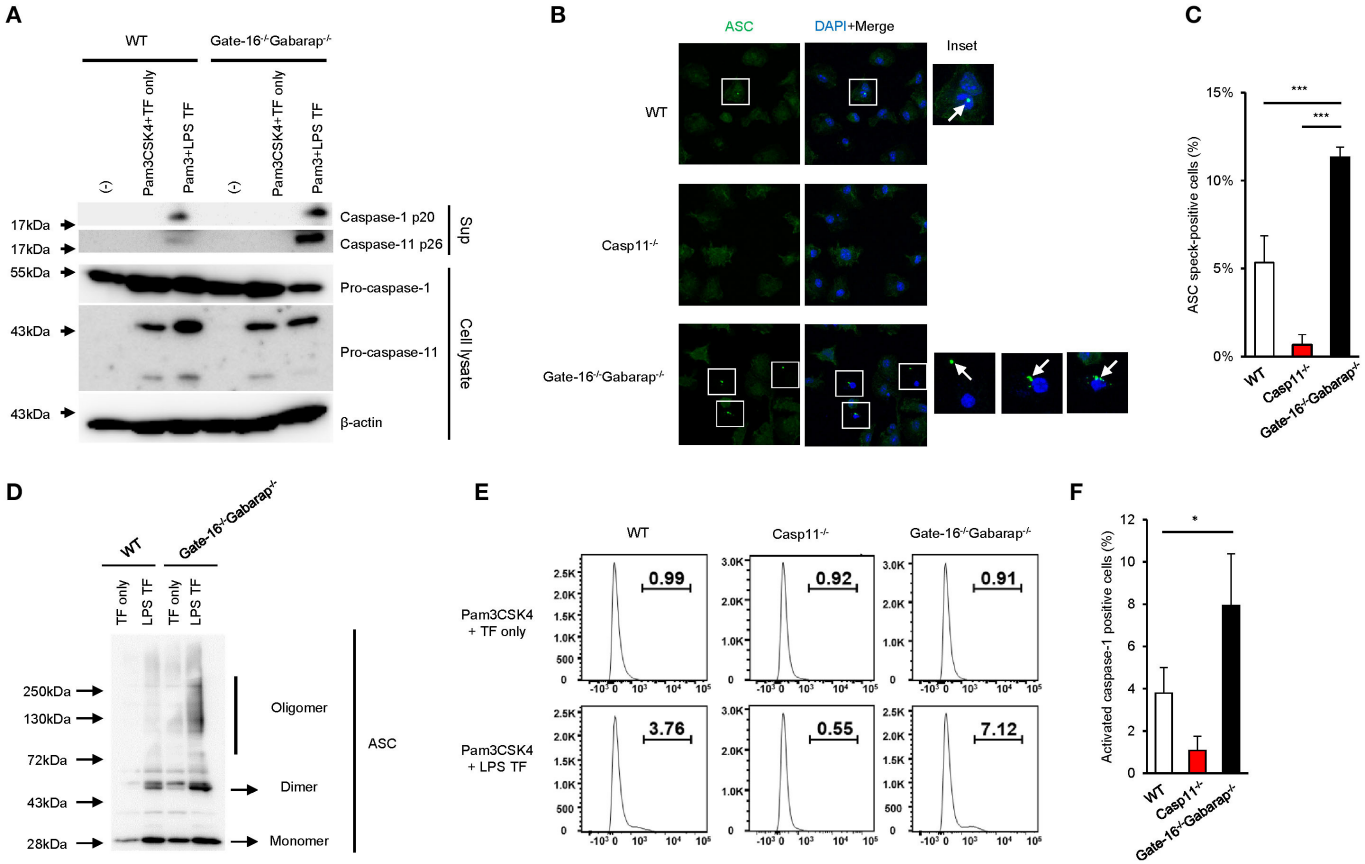
Enhanced caspase-11-mediated signaling pathways in *Gate-16*^−/−^*Gabarap*^−/−^ macrophages. **(A)** Western blot analysis of cleaved caspase-1 (p20) and caspase-11 (p26) in cell supernatants, and of pro-caspase-1 and pro-caspase-11 and Actin (loading control) in cell extracts of wild-type (WT) and *Gate-16*^−/−^*Gabarap*^−/−^ BMDMs primed with Pam3CSK4 followed by LPS transfection for 16 h. **(B)** Fluorescence confocal microscopy of wild-type (WT), Casp11^−/−^, and *Gate-16*^−/−^*Gabarap*^−/−^ BMDMs primed with Pam3CSK4 followed by transfection of LPS for 6 h and immunostained for ASC (green). The nucleus was stained with DAPI (blue). Squares show cells with ASC speck. Arrow indicates ASC speck itself. Scale bars correspond to 20 μm. **(C)** Percentage of ASC speck-positive wild-type (WT), Casp11^−/−^ and *Gate-16*^−/−^*Gabarap*^−/−^ BMDMs, as quantified by counting at least 100 cells per coverslip by confocal microscopy. **(D)** Oligomerization assay of ASC in cell extracts of wild-type (WT) and *Gate-16*^−/−^*Gabarap*^−/−^ BMDMs primed with Pam3CSK4 followed by LPS transfection for 6 h was analyzed by western blot. **(E,F)** Caspase-1 Pam3CSK4 primed wild-type (WT), Casp11^−/−^ and *Gate-16*^−/−^*Gabarap*^−/−^ BMDMs BMDMs were transfected with LPS for 4 h and added with FAM-FLICA to detect activated caspase-1 by flow cytometry **(E)** or by analysis for the quantification **(F)**. The data are representative of three independent experiments **(A,C,E)** and are combined data of more than three independent experiments **(C,F)**. ^*^P < 0.05, ^***^P < 0.001, two-tailed *t*-test.

Next, we assessed the intracellular signaling cascade for caspase-11 inflammasome activation in *Gate-16*^−/−^*Gabarap*^−/−^ cells. LPS transfection-mediated activation of caspase-11 in *Gate-16*^−/−^*Gabarap*^−/−^ cells was markedly augmented compared with wild-type cells (Figure 2A). Furthermore, the rate of *Gate-16*^−/−^*Gabarap*^−/−^ cells with caspase-11-dependent ASC speck formation in response to LPS transfection was significantly higher compared with wild-type cells (Figures 2B,C) and ASC oligomer formation in *Gate-16*^−/−^*Gabarap*^−/−^ cells was greater in response to LPS transfection than in wild-type cells (Figure 2D). Moreover, downstream of the caspase-11-ASC axis, the cleavage and activation of caspase-1 in LPS transfected *Gate-16*^−/−^*Gabarap*^−/−^ cells were also significantly increased compared with wild-type cells, whereas the total amounts of caspase-1 and caspase-11 proteins were comparable between wild-type and *Gate-16*^−/−^*Gabarap*^−/−^ cells (Figures 2A–F), indicating that hyperactivation of caspase-11 leads to upregulated ASC and caspase-1 activation, and enhanced IL-1β production and pyroptosis. In addition, OMV treatment- or *C. koseri* infection-induced release of caspase-11 and cleaved caspase-1 into supernatant was increased in *Gate-16*^−/−^*Gabarap*^−/−^ macrophages (Supplementary Figures 2A,B). In contrast, expression of NLRP3 and Pam3CSK4-induced pro-IL-1β production were comparable between wild-type and *Gate-16*^−/−^*Gabarap*^−/−^ cells (Supplementary Figure 2C), suggesting that Gate-16/Gabarap deficiency does not influence the priming induced expression of pro-IL-1β and NLRP3. Taken together, these results demonstrate that GABARAP subfamily proteins downregulate caspase-11 inflammasome activation.

### High Mortality in *Gate-16*^−/−^*Gabarap*^−/−^ Mice Following Sublethal LPS Challenge

**Figure 3:**
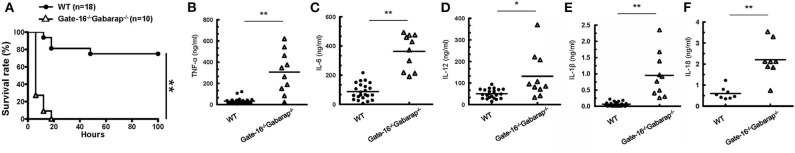
High mortality in *Gate-16*^−/−^*Gabarap*^−/−^ mice during LPS-induced sepsis. **(A)** Wild-type (WT: *n* = 18), and *Gate-16*^−/−^*Gabarap*^−/−^ (*n* = 10) mice were i.p. injected with 10 mg/kg body weight of poly(I:C) and then 7 h later i.p. injected with LPS (0.1 mg/kg body weight). Morbidity and mortality were observed for 100 h at 6 h intervals. **(B–F)** Wild-type and *Gate-16*^−/−^*Gabarap*^−/−^ mice were i.p. injected with 10 mg/kg body weight of poly(I:C) and then 7 h later i.p. injected with LPS (0.1 mg/kg body weight). Sera were taken 3 h after LPS injection from WT (*n* = 22) or *Gate-16*^−/−^*Gabarap*^−/−^ (*n* = 10) mice for TNF-α **(B)**, IL-6 (**C**), IL-12 **(D)**, and IL-1β **(E)** or from WT (*n* = 8) or *Gate-16*^−/−^*Gabarap*^−/−^ (*n* = 8) mice from IL-18 **(F)** serum concentrations were measured by ELISA. All data are combined data of three independent experiments. Log-rank test **(A)** and two-tailed Student *t*-test **(B–F)** ^*^P < 0.05 and ^**^P < 0.01.

We next examined the physiological relevance of the GABARAP subfamily-mediated negative regulation of non-canonical inflammasome activation. As previously reported (18, 19), mice primed with the TLR3 agonist poly(I:C) exhibited LPS-triggered inflammation and mortality in a caspase-11-dependent manner (Supplementary Figures 3A,B). After poly(I:C) priming, wild-type and *Gate-16*^−/−^*Gabarap*^−/−^ mice were intraperitoneally challenged with a low dose of LPS and monitored for survival (Figure 3A). Although 80% of wild-type mice survived, all *Gate-16*^−/−^*Gabarap*^−/−^ mice died within 20 h post-LPS injection (Figure 3A). Next, we compared the levels of inflammatory cytokines in sera between wild-type and *Gate-16*^−/−^*Gabarap*^−/−^ mice following LPS challenge (Figures 3B–F). Levels of IL-1β and IL-18, TNF-α, IL-6, and IL-12 in the sera of LPS-injected *Gate-16*^−/−^*Gabarap*^−/−^ mice were significantly higher compared with wild-type mice (Figures 3B–F), suggesting hyper caspase-11-dependent inflammation in *Gate-16*^−/−^*Gabarap*^−/−^ mice following LPS challenge. LPS lethality can be driven *via* TLR-induced TNF-α/IFN-β and caspase-8 (46). Therefore, we examined the role of TLR4 in the high sensitivity to LPS in *Gate-16*^−/−^*Gabarap*^−/−^ mice (Supplementary Figures 3C–F). When wild-type mice were treated with TAK-242, a potent inhibitor of TLR4 signaling (47), and subsequently challenged with intraperitoneal LPS injection alone, LPS infection-induced mortality and elevation of proinflammatory cytokines were profoundly inhibited (Supplementary Figures 3C,D). Next we treated *Gate-16*^−/−^*Gabarap*^−/−^ mice with the dose of TAK-242, and challenged the mice with sublethal LPS injection after poly(I:C) priming (Supplementary Figures 3E,F). Mortality and levels of proinflammatory cytokines in sera from *Gate-16*^−/−^*Gabarap*^−/−^ mice in the absence or presence of TAK-242 were comparable (Supplementary Figures 3E,F), indicating that TLR4 is dispensable for the hyper sensitivity to the polyIC/LPS-induced septic shock in *Gate-16*^−/−^*Gabarap*^−/−^ mice. Taken together, these data indicate that GABARAP autophagy proteins are important for the suppression of caspase-11-dependent sepsis.

### GABARAPs Specifically Down-Regulate GBP2-Dependent Caspase-11 Inflammasome

**Figure 4:**
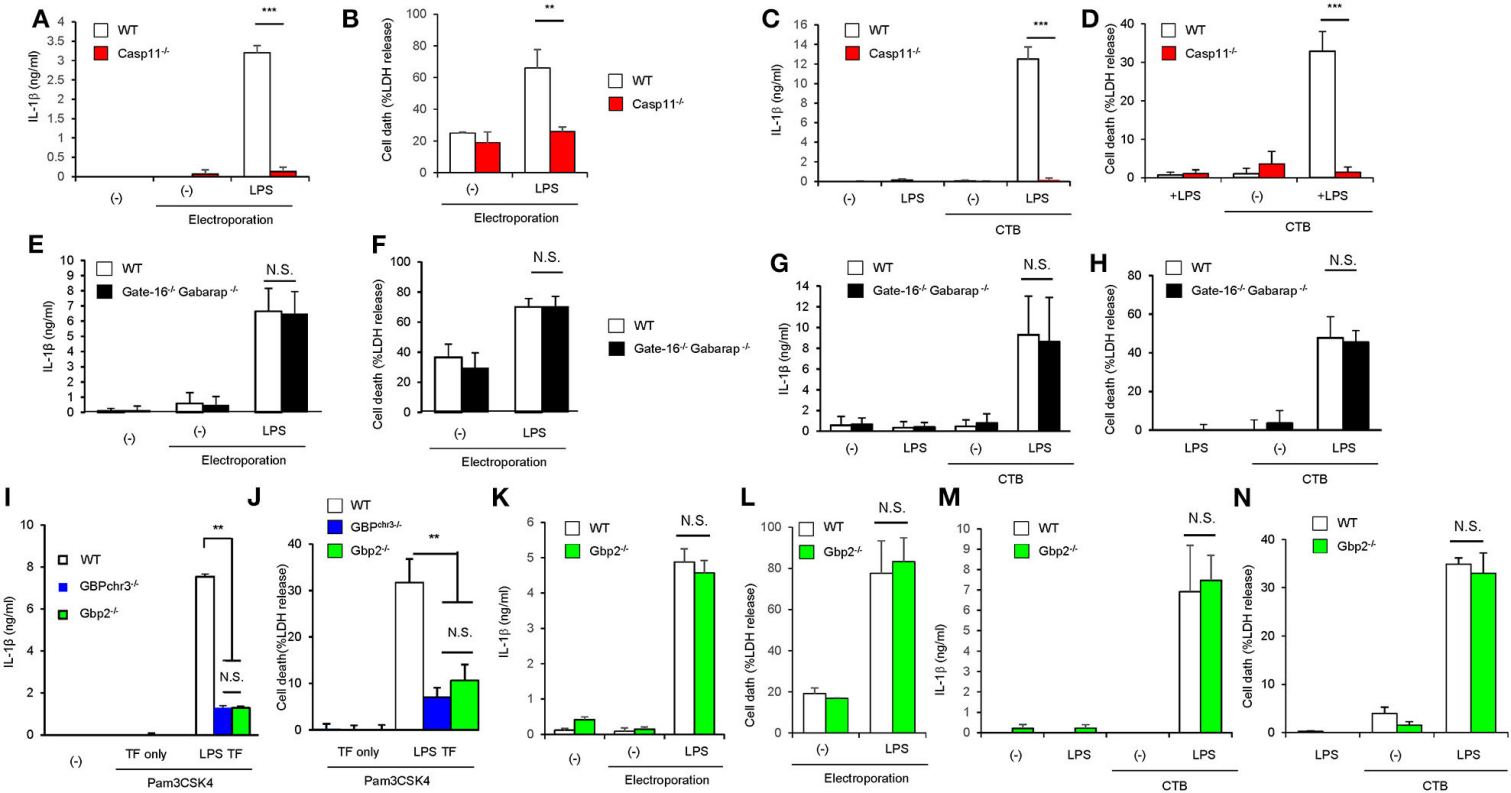
Enhanced activation of caspase-11 inflammasome in *Gate-16*^−/−^*Gabarap*^−/−^ macrophages is associated with the GBP2 dependency. **(A,B)** Release of IL-1β **(A)** and LDH **(B)** from Pam3CSK4-primed wild-type (WT) or *Casp11*^−/−^ BMDMs were electroporated with LPS and collected after 16 h. **(C,D)** Release of IL-1β **(C)** and LDH **(D)** from Pam3CSK4-primed wild-type (WT) and *Casp11*^−/−^ BMDMs were treated with LPS and CTB (LPS+CTB) for 16 h. **(E,F)** Release of IL-1β **(E)** and LDH **(F)** from Pam3CSK4-primed wild-type (WT) and *Gate-16*^−/−^*Gabarap*^−/−^ BMDMs were electroporated with LPS and collected after 16 h. **(G,H)** Release of IL-1β **(G)** and LDH **(H)** from Pam3CSK4-primed wild-type (WT) and *Gate-16*^−/−^*Gabarap*^−/−^ BMDMs were treated with LPS+CTB for 16 h. **(I,J)** Release of IL-1β **(B)** or LDH **(C)** from wild-type (WT), *GBPchr3*^−/−^, and *Gbp2*^−/−^ BMDMs primed with Pam3CSK4 followed by LPS transfection for 16 h. **(K,L)** Release of IL-1β **(K)** and LDH **(L)** from Pam3CSK4-primed wild-type (WT) and *Gbp2*^−/−^ BMDMs were electroporated with LPS and collected after 16 h. **(M,N)** Release of IL-1β **(M)** and LDH **(N)** from Pam3CSK4-primed wild-type (WT) and *Gbp2*^−/−^ BMDMs were electroporated with LPS and collected after 16 h. The data are combined data of more than three independent experiments **(A–N)**. ^**^P < 0.01, ^***^P < 0.001, and N.S., not significant, two-tailed Student *t*-test.

We next explored the mechanism of how GABARAP deficiency specifically hyperactivates caspase-11 in response to LPS transfection. Caspase-11 directly sense cytosolic LPS (18, 19). In addition to LPS introduction by liposome, cholera toxin fragment B (CTB) and electroporation-mediated LPS introduction are shown to induce caspase-11-dependent immune response (8, 19) (Figures 4A–D). Therefore, we first examined various methods of LPS introduction into the cytosol and compared the subsequent caspase-11-mediated responses between wild-type and DKO macrophages (Figures 4E–H). Notably, although the electroporation of LPS led to caspase-11-dependent IL-1β production and pyroptosis (Figures 4A,B), the caspase-11-dependent responses in *Gate-16*^−/−^*Gabarap*^−/−^ macrophages were not observed in response to LPS electroporation (Figures 4E,F). Furthermore, CTB-mediated LPS introduction (CTB-LPS) resulted in caspase-11-dependent IL-1β production (Figures 4C,D), whereas *Gate-16*^−/−^*Gabarap*^−/−^ cells showed normal caspase-11-mediated responses in response to CTB-mediated LPS introduction (Figures 4G,H), indicating that the GABARAP deficiency does not affect all of the caspase-11-dependent responses. GBPs are required for caspase-11 activation and immune responses to LPS transfection (Figures 4I,J). *Gbp2*^−/−^ macrophages showed defective IL-1β production and pyroptosis similar to *GBPchr3*^−/−^ cells that lack all GBPs on chromosome 3 such as *Gbp1, Gbp2, Gbp3, Gbp5*, and *Gbp7* (33, 36) (Figures 4I,J), indicating the major role of GBP2 in LPS transfection-induced activation of the caspase-11 inflammasome (Supplementary Figures 4A–D). When the IL-1β production and cell death in *Gbp2*^−/−^ macrophages in response to CTB-LPS or LPS electroporation were examined, the caspase-11-dependent responses in *Gbp2*^−/−^ cells were normal compared with wild-type cells (Figures 4K–N), suggesting that the GBP2 deficiency as likely as the GABARAP deficiency only affect the caspase-11-dependent responses induced by liposomal transfection of LPS but not by CTB-LPS or LPS electoporation. Moreover, canonical inflammasome activation by LPS+ATP stimulation was not affected in *Gbp2*^−/−^ macrophages as well as *Gate-16*^−/−^*Gabarap*^−/−^ cells (Supplementary Figures 4E–G). The amounts of cytosolic and non-cytosolic LPS were comparable between wild-type and *Gate-16*^−/−^*Gabarap*^−/−^ cells (Supplementary Figure 4H), suggesting that LPS entry into the cytosol is not enhanced by the GABARAP deficiency. Collectively, the hypersensitivity to LPS in *Gate-16*^−/−^*Gabarap*^−/−^ cells was observed in parallel with GBP2 dependency.

### GBP2 Aggregation by GABARAP Deficiency Enhances Caspase-11-Induced Response

**Figure 5:**
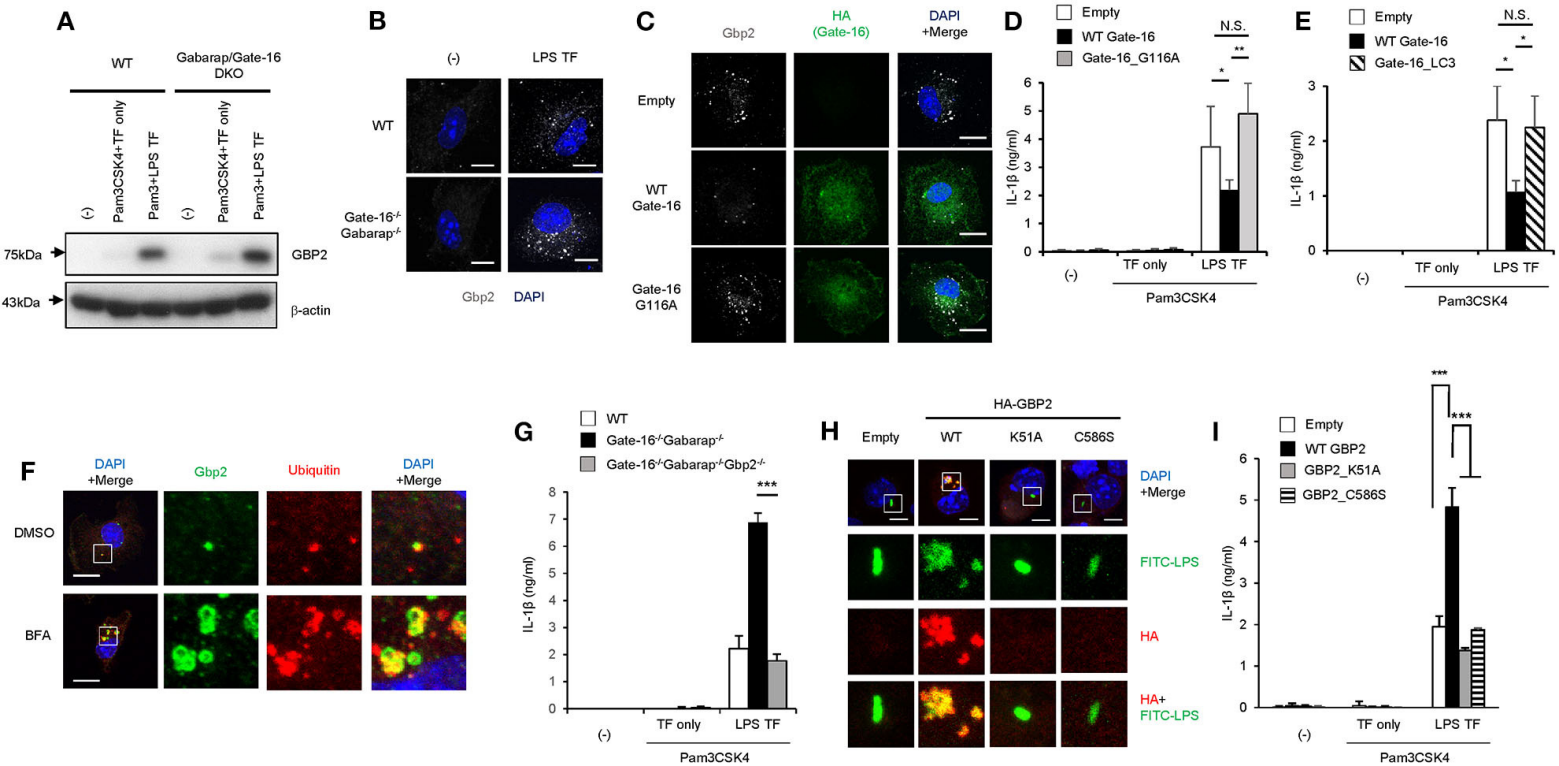
GBP2-dependent enhanced caspase-11-dependent responses by *Gate-16*/Gabarap deficiency. **(A)** Immunoblots of the indicated protein expressions in WT and *Gate-16*^−/−^*Gabarap*^−/−^ BMDMs primed with Pam3CSK4 followed by LPS or mock transfection for 16 h. **(B)** Fluorescence confocal microscopy of wild-type (WT) and *Gate-16*^−/−^*Gabarap*^−/−^ BMDMs stimulated by LPS transfection for 6 h and immunostained for GBP2 (white). The nucleus was stained with DAPI (blue). Scale bars correspond to 5 μm. **(C)** Fluorescence confocal microscopy of *Gate-16*^−/−^*Gabarap*^−/−^ BMDMs stably expressing HA-tagged wild-type Gate-16 (WT Gate-16), the G116A mutant (G116A Gate-16) and empty vectors. The cells were stimulated by LPS transfection for 6 h and immunostained for GBP2 (white) and HA (green). The nucleus was stained with DAPI (blue). Scale bars correspond to 10 μm. **(D,E)** Release of IL-1β from *Gate-16*^−/−^*Gabarap*^−/−^ BMDMs stably expressing wild-type Gate-16 (WT Gate-16), empty vector, the G116A mutant (Gate-16_G116A) **(D)** or the defective Arf1 binding mutant (Gate-16_LC3) **(E)**. The cells were primed with Pam3CSK4 and subsequently stimulated by LPS transfection for 16 h. **(F)** Fluorescence confocal microscopy of IFN-β-stimulated wild-type BMDMs untreated or treated with Brefeldin A (BFA, 35 μM) for 1 h and immunostained for GBP2 (green) and ubiqutin (red). The nucleus was stained with DAPI (blue). Scale bars correspond to 5 μm. **(G)** Release of IL-1β **(G)** from wild-type (WT), *Gate-16*^−/−^*Gabarap*^−/−^ and *Gate-16*^−/−^*Gabarap*^−/−^*Gbp2*^−/−^ BMDMs primed with Pam3CSK4 followed by LPS transfection for 16 h. **(H)** Fluorescence confocal microscopy of Pam3CSK4-primed *Gate-16*^−/−^*Gabarap*^−/−^*Gbp2*^−/−^ BMDMs stably expressing HA-tagged wild-type GBP2 (WT), the GTPase-inactive mutant (K51A), the non-isoprenylated mutant (C586S) and empty vectors, subsequently stimulated by FITC-LPS (green) transfection for 2 h and immunostained for HA (red). The nucleus was stained with DAPI (blue). Scale bars correspond to 5 μm. **(I)** Release of IL-1β from *Gate-16*^−/−^*Gabarap*^−/−^*Gbp2*^−/−^ BMDMs stably expressing HA-tagged wild-type GBP2 (WT GBP2), the GTPase-inactive mutant (GBP2_K51A), the non-isoprenylated mutant (GBP2_C586S) and empty vectors. The cells were primed with Pam3CSK4 and subsequently stimulated by LPS transfection for 16 h. The data are representative of three independent experiments **(A–C,F,H)** and are combined data of more than three independent experiments **(D,E,G,I)**. ^*^P < 0.05, ^**^P < 0.01, ^***^P < 0.001, and N.S., not significant, two-tailed Student *t*-test.

We then explored how GBP2 is involved in the hyperactivation of caspase-11 inflammasomes in *Gate-16*^−/−^*Gabarap*^−/−^ macrophages. Upon LPS transfection, GBP2 was normally induced and expressed in *Gate-16*^−/−^*Gabarap*^−/−^ macrophages compared to wild-type cells (Figure 5A). Moreover, Pam3CSK-induced expression of GBP2 was also intact in *Gate-16*^−/−^*Gabarap*^−/−^ macrophages (Supplementary Figure 2C), indicating that Gate-16/Gabarap deficiency does not impact priming- or LPS transfection-induced GBP2 expression. Because GBP2 was previously shown to uniformly localize in dot-like structures in the cytosol in fibroblasts (42), we investigated the localization of GBP2 in *Gate-16*^−/−^*Gabarap*^−/−^ macrophages (Figure 5B and Supplementary Figures 5A,B). Notably, we found that *Gate-16*^−/−^*Gabarap*^−/−^ macrophages contained significantly higher numbers of GBP2 puncta with higher total areas, compared with wild-type cells (Figure 5B and Supplementary Figures 5A,B). We previously showed that GBPs were uniformly localized in the cytosol dependent on lipidated Gate-16 in fibroblasts (42). When wild-type Gate-16 was re-constituted in *Gate-16*^−/−^*Gabarap*^−/−^ macrophages, the aggregations of GBP2 were resolved in terms of cell number and size (Figure 5C and Supplementary Figures 5C,D). Moreover, LPS transfection-induced IL-1β production in wild-type Gate-16-transduced *Gate-16*^−/−^*Gabarap*^−/−^ macrophages was significantly reduced (Figure 5D). Furthermore, ectopic expression of Gabarap in *Gate-16*^−/−^*Gabarap*^−/−^ cells significantly decreased IL-1β production albeit less efficiently than that of Gate-16 (Supplementary Figure 5E). In contrast, re-introduction of the G116A mutant of Gate-16, which is defective for C-terminal lipidation (42), into *Gate-16*^−/−^*Gabarap*^−/−^ cells failed to dissolve GBP2 aggregation or reduce IL-1β production (Figures 5C,D and Supplementary Figures 5C,D), demonstrating that normal GBP2-dependent caspase-11-mediated immune responses require the C-terminal lipidation of Gate-16. We previously demonstrated that GABARAP autophagy proteins regulate uniform distribution of GBP-containing vesicles in a manner dependent on Arf1, a Golgi-localizing small GTPase which is essential for COPI vesicle formation from Golgi membranes (42, 48). Gate-16 but not LC3b interacts with Arf1 (42). An N-terminal portion of Gate-16 determines the specific interaction with Arf1, and the amino acid sequences of GABARAP subfamily and those of LC3 subfamily are distinct (42). The Gate-16 mutant (Gate-16_LC3), in which the Arf1 binding portion is replaced with that of LC3b, not only fails to interact with Arf1 but also does not restore uniform distribution of GBP-containing vesicles in *Gate-16*^−/−^*Gabarap*^−/−^*Gabarapl1*^−/−^ cells (42). Therefore, to test whether Arf1 is involved in the excess caspase-11-dependent response in *Gate-16*^−/−^*Gabarap*^−/−^ cells, we examined whether ectopic expression of the Gate-16_LC3 in *Gate-16*^−/−^*Gabarap*^−/−^ cells restores the caspase-11-dependent response (Figure 5E). We found that reconstitution of the Gate-16_LC3 mutant in *Gate-16*^−/−^*Gabarap*^−/−^ macrophages also failed to reduce IL-1β production (Figure 5E), suggesting the involvement of Arf1 in the excess caspase-11-dependent immune response. When wild-type macrophages were treated with Brefeldin A, an inhibitor of Arf1 (49), GBP2 were mislocalized and aggregated (Figure 5F), reminiscent of that in *Gate-16*^−/−^*Gabarap*^−/−^ cells (Figure 5B). Moreover, the GBP2 aggregates contained ubiquitin (Figure 5F), which was reminiscent of the phenotypes of cells lacking GABARAPs or some of Atg proteins (42). Thus, these data together our previous study suggest that Arf1 is involved in Gate-16-mediated uniform GBP2 distribution (42).

To directly test the role of GBP2 in hyperactivated caspase-11 inflammasomes in *Gate-16*^−/−^*Gabarap*^−/−^ macrophages, we generated *Gate-16*^−/−^*Gabarap*^−/−^*Gbp2*^−/−^ mice and examined the macrophage response to LPS transfection (Figure 5G and Supplementary Figures 5F–H). IL-1β release in LPS-transfected *Gate-16*^−/−^*Gabarap*^−/−^ or *Gate-16*^−/−^*Gabarap*^−/−^*Gbp2*^−/−^ macrophages showed that IL-1β release in *Gate-16*^−/−^*Gabarap*^−/−^*Gbp2*^−/−^ cells were significantly lower than that in *Gate-16*^−/−^*Gabarap*^−/−^ cells in a time-dependent manner (Figure 5G and Supplementary Figure 5F). Cell death in LPS transfected *Gate-16*^−/−^*Gabarap*^−/−^*Gbp2*^−/−^ macrophages were lower than in *Gate-16*^−/−^*Gabarap*^−/−^ cells (Supplementary Figure 5G). Furthermore, time-dependent *C. koseri*-infected IL-1β production in *Gate-16*^−/−^*Gabarap*^−/−^*Gbp2*^−/−^ cells was significantly impaired when compared to *Gate-16*^−/−^*Gabarap*^−/−^ cells (Supplementary Figure 5H), suggesting an important role of GBP2 in the enhanced caspase-11-mediated responses. However, *Gate-16*^−/−^*Gabarap*^−/−^*Gbp2*^−/−^ cells still displayed more IL-1β production than wild-type cells (Supplementary Figure 5H). GBPs on chromosome 3 are important for *C. koseri*-induced IL-1β production (33). Moreover, *Gbp2*^−/−^ cells showed partial reduction of *C. koseri*-induced IL-1β production (Supplementary Figure 5I), suggesting that GBPs other than GBP2 may contribute to the IL-1β production in *Gate-16*^−/−^*Gabarap*^−/−^*Gbp2*^−/−^ cells. GBP2 is an enzymatically active GTPase that is isoprenylated at the C-terminus (50). We next assessed whether the GTPase activity and isoprenylation of GBP2 were involved in the enhanced IL-1β production of *Gate-16*^−/−^*Gabarap*^−/−^ macrophages (Figures 5H,I). The introduction of wild-type GBP2, but not GTPase inactive K51A or non-isoprenylated C586S mutants, into *Gate-16*^−/−^*Gabarap*^−/−^*Gbp2*^−/−^ cells resulted in GBP2 aggregation and the upregulation of IL-1β production (Figures 5H,I), suggesting GBP2 requires both GTPase activity and isoprenylation for the enhanced caspase-11-dependent responses in *Gate-16*^−/−^*Gabarap*^−/−^ macrophages. Notably, we found that the transfected FITC-LPS was co-localized with wild-type GBP2 but not the K51A or C586S mutants (Figure 5H), indicating that both GTPase activity and isoprenylation of GBP2 are important for the GBP2 co-localization with LPS. Collectively, these results demonstrate that GBP2 plays a critical role in the elevated caspase-11-induced immune response in *Gate-16*^−/−^*Gabarap*^−/−^ macrophages in response to LPS transfection and bacterial infection.

### Lack of GBP2 Attenuates Caspase-11-Mediated Excessive Inflammation in *Gate-16*^−/−^*Gabarap*^−/−^ Mice in Sepsis Models

**Figure 6:**
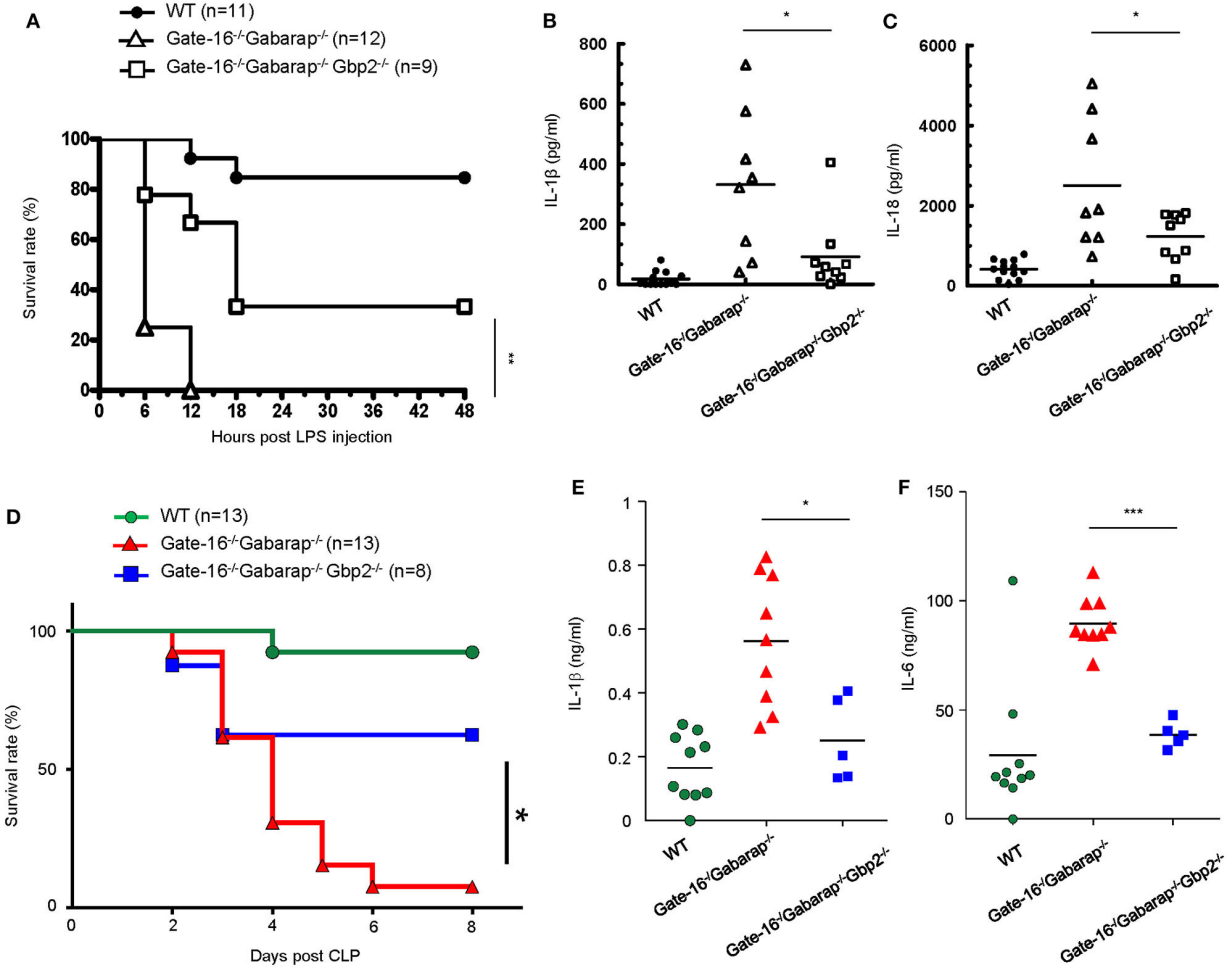
GBP2-dependent high mortality in *Gate-16*^−/−^*Gabarap*^−/−^ mice during sepsis. **(A)** Wild-type (WT: *n* = 11), *Gate-16*^−/−^*Gabarap*^−/−^ (*n* = 12), and *Gate-16*^−/−^*Gabarap*^−/−^ Gbp2^−/−^ (*n* = 9) mice were i.p. injected with 10 mg/kg body weight of poly(I:C) and then 7 h later i.p. injected with LPS (0.1 mg/kg body weight). Morbidity and mortality were observed for 48 h at 6 h intervals. **(B,C)** Wild-type (WT), *Gate-16*^−/−^*Gabarap*^−/−^, and *Gate-16*^−/−^*Gabarap*^−/−^Gbp2^−/−^ mice were i.p. injected with 10 mg/kg body weight of poly(I:C) and then 7 h later i.p. injected with LPS (0.1 mg/kg body weight). Sera were taken 3 h after LPS injection from WT (*n* = 13), *Gate-16*^−/−^*Gabarap*^−/−^ (*n* = 8) or *Gate-16*^−/−^*Gabarap*^−/−^Gbp2^−/−^ (n = 9) mice for IL-1β **(B)** and IL-18 **(C)** serum concentrations were measured by ELISA. **(D)** CLP was performed in wild-type (WT: *n* = 13), *Gate-16*^−/−^*Gabarap*^−/−^ (*n* = 13), and *Gate-16*^−/−^*Gabarap*^−/−^ Gbp2^−/−^ (*n* = 8) mice. Morbidity and mortality were observed for 8 days at 1 day intervals. **(E,F)** CLP was performed in wild-type (WT; *n* = 10), *Gate-16*^−/−^*Gabarap*^−/−^ (*n* = 9) and *Gate-16*^−/−^*Gabarap*^−/−^Gbp2^−/−^ (*n* = 5) mice. Peritoneal fluids were taken 16 h after CLP from the mice for IL-1β **(E)** and IL-6 **(F)** serum concentrations were measured by ELISA. The data are combined data of more than three independent experiments **(A–F)**. Log-rank test **(A,D)** and two-tailed Student *t*-test **(B,C,E,F)** ^*^P < 0.05, ^**^P < 0.01 and ^***^P < 0.001.

Finally we examined the physiological relevance of the GABARAP subfamily-mediated negative regulation of GBP2-dependent non-canonical inflammasome activation. As previously reported (51), mice primed with poly(I:C) exhibited LPS-triggered inflammation and mortality dependent on GBP2 and caspase-11 (Supplementary Figures 3A,B). Moreover, *Gate-16*^−/−^*Gabarap*^−/−^*Gbp2*^−/−^ mice were more resistant to low dose LPS challenge (Figure 6A) and exhibited lower levels of IL-1β and IL-18 in the sera (Figures 6B,C) compared with *Gate-16*^−/−^*Gabarap*^−/−^ mice. Next we tested the physiological significance of Gate-16 and Gabarap for inhibition of GBP2-dependent caspase-11 inflammasome activation during polymicrobial sepsis in the CLP model (52) (Figure 6D). Compared with wild-type mice, *Gate-16*^−/−^*Gabarap*^−/−^ mice showed increased mortality during the CLP and markedly higher production of IL-1β and IL-6 in the peritoneal fluids (Figures 6D–F). In contrast, the elevation of cytokines and mortality was significantly ameliorated in *Gate-16*^−/−^*Gabarap*^−/−^*Gbp2*^−/−^ mice (Figures 6D–F). Taken together, these results suggest that GABARAP subfamily members specifically and physiologically downregulate GBP2-dependent caspase-11-induced innate immune responses to prevent septic shock.

## Discussion

In this study, we demonstrated that deficiency of the GABARAP subfamily proteins such as Gate-16 and Gabarap enhances activation of caspase-11 inflammasome in response to specific LPS stimulation. We found how LPS was prepared for the stimulation of *Gate-16*^−/−^*Gabarap*^−/−^ or *Gbp2*^−/−^ macrophages determined whether caspase-11 inflammasome activation was upregulated or downregulated, respectively. Regarding the caspase-11-dependent response, the opposite phenotypes of *Gate-16*^−/−^*Gabarap*^−/−^ and *Gbp2*^−/−^ cells were only observed for LPS transfection, *C. koseri* infection and OMV stimulation but not for other stimuli such as LPS electroporation and CTB-LPS treatment. Electroporation by high electronic pulse forms pores on the plasma membranes (53), allowing LPS to be directly transferred into the cytoplasm. CTB physiologically transports cholera toxin A fragment from the plasma membrane into the cytoplasm (54). Thus, the co-internalization of LPS and CTB into the cytosol may result in the cytosolic exposure of LPS and subsequent activation of caspase-11 (7). However, LPS is included in liposomes or membranous vesicles in the case of LPS transfection, or bacterial infection and OMV (44). The liposomally transfected LPS may be in membranous structures as likely as OMV that is naturally generated by Gram negative bacteria (44). Whether CTB- or electroporation-mediated LPS transfer physiologically happens or not is uncertain, however, infection of *Vibrio cholerae*, which naturally possesses CTB (55), might activate caspase-11 inflammasome in GBP2-dependent and -independent pathways (33).

We found that *Gate-16*^−/−^*Gabarap*^−/−^ macrophages showed elevated immune responses to GBP2-dependent non-canonical NLRP3 inflammasome-dependent stimuli but not to canonical NLRP3 inflammasome-dependent stimulation. For canonical NLRP3 inflammasomes, various inhibitory mechanisms were previously reported. NLRP3 mRNA transcription is inhibited by Ahr and miR-233 (20, 21) and NLRP3 mRNA translation is prevented by TTP (22). NLRP3 protein is ubiquitinated or nitrosylated by ARDH2 and nitric oxide, respectively (23, 24). NLRP3 sensing of ROS is modulated by TRIM30, Tim-3 and Nrf2 (25–27). Moreover, the NLRP3 inflammasome is inhibited by type I interferon and IKKα by regulating IL-1β mRNA transcription and ASC proteins, respectively (28, 29). Given that NLRP3 is shared by both the canonical and caspase-11 inflammasomes, these negative regulators might also suppress LPS-mediated excessive inflammation and septic shock. However, little is known about which inhibitory molecules are specific for caspase-11 inflammasomes, except for stearoyl lysophosphatidylcholine (56). Our study demonstrates that GABARAPs are specifically involved in caspase-11 inflammasomes upstream of caspase-11, but not in the canonical NLRP3, Aim2, and NLRC4 inflammasomes. Thus, GABARAPs are specific negative regulators of the caspase-11 inflammasome. However, a previous study showed that *Gabarap*^−/−^ macrophages displayed increased responses of canonical NLRP3 inflammasome and high mortality after CLP (30). On the other hand, responses of canonical NLRP3 inflammasome in our *Gabarap*^−/−^ macrophages were normal. The discrepancy might be caused by different strategies for generation of *Gabarap*^−/−^ mice or different genetic background of the mice between the two studies. To check whether the phenotype found in a gene-targeted cells is restored by reintroduction of the gene is important to prove that the phenotype is caused by loss of the gene. In the present study, we confirmed that the liposomally transfected LPS-mediated increased IL-1β production in *Gate-16*^−/−^*Gabarap*^−/−^ macrophages was restored by reintroduction of Gabarap to some extent, indicating the phenotype is due to loss of Gabarap. Further studies to compare the two different *Gabarap*^−/−^ mouse lines will clarify the involvement of Gabarap in the negative regulation of canonical and non-canonical NLRP3 inflammasome activation. In addition, Nedd4 is very recently shown to mediate caspase-11 degradation by K48-linked polyubiquitination (32), indicating that caspase-11 activity might be tightly controlled by the protein amounts and uniform distribution by Nedd4 and GABARAPs, respectively. Furthermore, SERPINB1 plays a role in inhibiting LPS-induced inflammasome activation (31). Thus, reports regarding inhibitory mechanisms on caspase-11 inflammasome activation are currently growing.

Atg12-deficient macrophages exhibited the over-activation of canonical and caspase-11 inflammasomes. A deficiency in GABARAPs resulted in the enhanced activation of caspase-11 inflammasomes whereas no effect was observed for the canonical inflammasome. However, a deficiency of LC3s led to the enhanced activation of canonical inflammasomes but not caspase-11 inflammasomes. Thus, downstream of Atg12, GABARAPs, and LC3s play different roles in the suppression of NLRP3 inflammasome activation. Atg12-deficient or *Lc3a*^−/−^*Lc3b*^−/−^ macrophages contained more damaged mitochondria than wild-type cells in response to LPS plus ATP stimulation as previously reported (45). The accumulation of damaged mitochondria is caused by an impairment in autophagy termed mitophagy due to lack of Atg12 or LC3s (57). In contrast, the formation of GBP2 aggregates due to lack of Gate-16 and Gabarap is the direct cause of caspase-11 over-activation and is independent of autophagy because GBP aggregation was observed only in cells lacking Atg3, Atg5, Atg7, or Atg16L1, all of which are essential for Atg8 lipidation, but not in cells lacking Atg9, Atg14, or FIP200, all of which are essential for autophagy (39, 42). Our current and previous study demonstrate that Arf1 inhibition by Brefeldin A in wild-type cell resulted in ubiquitin aggregates containing GBPs (42). Arf1 is a small GTPase important for generation of COPI-coated vesicles from Golgi during intra-Golgi transport (48). In addition, Gate-16 was originally identified as Golgi-associated ATPase enhance 16kD (58), whose function was not initially linked not with autophagy but only with intra-Golgi transport (59). Given that GBP2 is detected in microsomal fractions that also contain Golgi vesicles, Gate-16 (and also Gabarap) *via* interaction with Arf1 may regulate the generation of uniformly small size of Golgi-derived vesicles including GBP2 in an autophagy-independent fashion. Deficiency of the GABARAPs as likely as Arf1 inhibition might lead to failure of generation of the GBP2-containing vesicles, resulting in formation of large GBP2 aggregates which enhance GBP2-mediated caspase-11-depenendent response. Thus, our data indicate that, although the Atg12-LC3 subfamily axis suppresses canonical NLRP3 inflammasomes by autophagy (mitophagy), the Atg12-GABARAP subfamily axis negates GBP2-dependent activation of non-canonical NLRP3 inflammasomes in an autophagy-independent manner.

We also demonstrated that poly(I:C)-primed *Gate-16*^−/−^*Gabarap*^−/−^ mice were highly susceptible to low dose LPS-induced and polymicrobial septic shock. However, the increased mortality of LPS-injected *Gate-16*^−/−^*Gabarap*^−/−^ mice was not completely prevented by additional GBP2 deficiency. In this regard, TLR4 may not play a role in the elevated immune responses in *Gate-16*^−/−^*Gabarap*^−/−^ mice since the pharmacological TLR4 inhibition did not reduce high mortality and levels of proinflammatory cytokines in sera. On the other hand, it is possible that increased canonical inflammasome activation and its inflammation by Gabarap deficiency could contribute to the high mortality in a manner independent on GBP2, since Gabarap has been shown as a negative regulator of canonical NLRP3 inflammasome and prevents LPS-induced lethality (30, 60). Furthermore, we have found the physiological relevance of a negative regulatory mechanism for GBP2-dependent caspase-11 inflammasome activation, which is essential for the prevention of LPS-mediated and polymicrobial septic shock *in vivo*. OMVs deliver a number of membrane-bound antigens, OMV-based vaccines have attracted much attention (61). However, our current study indicates that we should be cautious of using OMV vaccine treatment in humans with a mutation in ATG16L1, in which GABARAPs are not lipidated and hence remain inactivated (62), because of the potential for strong non-inflammation responses and septic shock compared with normal individuals. Small compounds targeting the GTPase activity or the isoprenylation site of GBP2 might be helpful to attenuate the endotoxemia.

## Methods

### Mice, Bacteria and Cells

All C57BL/6N mice were obtained from SLC. *Gabarap*^−/−^, *Gabarapl1*^−/−^, *Gate-16*^−/−^, *Lc3a*^−/−^, *Lc3b*^−/−^, *GBPchr3*^−/−^, *Gbp2*^−/−^ mice, and LysM-Cre mice were used and described previously (42, 63, 64). *Casp1*^−/−^, *Casp11*^−/−^, *Atg12*^fl/fl^, *Aim2*^−/−^, and *Nlrc4*^−/−^ mice newly generated in this study (Supplementary Figure 6). All of wild-type or mutant mice were in C57BL/6N background. All animal experiments were performed with the approval of the Animal Research Committee of Research Institute for Microbial Diseases in Osaka University. *C. koseri* was used as described previously (42), and *Escherichia coli* BL21 strain was used. 293T cells (CRL-3216) and J774 cells (TIB-67) were obtained from the ATCC.

### Antibodies and Reagents

For immunoblot studies, antibodies against caspase-11 (C1354) and β-actin (A1978) were purchased from SIGMA. Antibody against caspase-1 (AG-20B-0042) was purchased from Adipogen. Antibodies against Gabarap (M135-3) and Gate-16 (18724-1-AP) were purchased from MBL or Proteintech. Antibody against GBP2 (sc-10588) was purchased from Santa Cruz. Antibody against ASC (04-147) was obtained from Millipore. TOM20 (11802-1-AP) was obtained from Proteintech. Antibody against β-COP (ab2899) were purchased from Abcam. Anti-Aim2 (ab93015) was purchased from Abcam. Anti-NLRC4 (06-1125) was purchased from Millipore.

For immunofluorescence studies, anti-GBP2 (11854-1-AP) were purchased from Proteintech. Anti-HA (MMS-101R) was purchased from Covance. Anti-caspase-11 (NB120-10454) was obtained from Novus. Anti-caspase-11 (C1354) and FITC-LPS (F3665) were obtained from Sigma. Anti-ASC (#67824) was obtained from Cell Signaling. Anti-ubiquitin mouse monoclonal antibody (FK2; MFK-004) was obtained from Nippon Biotest Labotatories. Anti-p62 (PM045) was obtained from MBL.

### Generation of *Casp-1*^−/−^, *Casp-11*^−/−^, *Atg12*^fl/fl^, *Aim2*^−/−^, and *Nlrc4*^−/−^ Mice by Cas9/CRISPR-Mediated Genome Editing

The T7-transcribed caspase-1, caspase-11, Atg12, Aim2, and NLRC4 gRNA PCR products, which were amplified by using KOD FX NEO (Toyobo) and the primers (Supplementary Figures 6A–E and Supplementary Table 1), were used as the subsequent generation of caspase-1, caspase-11, Atg12 Aim2, and NLRC4 gRNAs. And then, MEGAshortscript T7 (Life Technologies) was used for the generation of these gRNAs. Cas9 mRNA was generated by *in vitro* transcription (IVT) using mMESSAGE mMACHINE T7 ULTRA kit (Life technologies) and the template that was amplified by PCR using pEF6-hCas9-Puro and the primers T7Cas9_IVT_F and Cas9_R (65), and gel-purified. The synthesized gRNA and Cas9 mRNA were purified using MEGAclear kit (Life Technologies) and eluted in RNase-free water (Nacalai Tesque). To obtain *Casp1*^−/−^ or *Casp11*^−/−^ mice, C57BL/6N female mice (6 weeks old) were superovulated and mated to C57BL/6N stud males. Fertilized one-cell-stage embryos were collected from oviducts and injected into the pronuclei or the cytoplasm with 100 ng/μl Cas9 mRNA and 50 ng/μl each gRNA in accordance with a previous study as described previously (66). The injected live embryos were transferred into oviducts of pseudopregnant ICR females at 0.5 d post-coitus. The male pup harboring the mutation was mated to C57BL/6N female mice and tested for the germ line transmission. Heterozygous mice were intercrossed to generate homozygous *Casp1*^−/−^, *Casp11*^−/−^, *Aim2*^−/−^, or *Nlrc4*^−/−^ mice. Bone-marrow derived macrophages (BMDMs) from *Casp1*^−/−^, *Casp11*^−/−^, *Aim2*^−/−^, or *Nlrc4*^−/−^ mice lacked caspase-1 (Supplementary Figure 6F), caspase-11 (Supplementary Figure 6F), Aim2 (Supplementary Figure 6G) or NLRC4 proteins (Supplementary Figure 6H), respectively. To obtain *Gate-16*^−/−^*Gabarap*^−/−^*Gbp2*^−/−^ mice, *Gate-16*^−/−^*Gabarap*^−/−^ mice, and *Gbp2*^−/−^ mice were intercrossed. For generation of the targeting fragment for the floxed *Atg12* allele, the *Atg12* gene was isolated from genomic DNA that was extracted from C57BL/6N embryonic fibroblasts by PCR using KOD FX NEO (Toyobo) and primers (Supplementary Figure 6E and Supplementary Table 1). The targeting fragment was constructed from a 0.5-kb fragment of *Atg12* genomic DNA containing exon 2 and *lox*P site-containing 0.5-kb subfragments using restriction enzymes in pBluescript. The vectors were amplified and co-injected into the embryos with the Cas9-encoding mRNA and Atg12flox1/Atg12flox2_gRNA to obtain *Atg12*^fl/+^ pups. *Atg12*^fl/+^ mice were further crossed with LysM-Cre mice to generate LysM-Cre *Atg12*^fl/fl^ mice (*Atg12*^Δmyeloid^ mice). BMDMs from *Atg12*^Δmyeloid^ mice lacked Atg12 protein, which is stably conjugated with Atg5 (Supplementary Figure 6I).

### Cell Culture and Bacterial Infection

293T cells or J774 cells were maintained in DMEM medium (Nacalai Tesque) or RPMI medium (Nacalai tesque) including 10% heat-inactivated FBS (JRH Bioscience), 100 U/ml penicillin (Nacalai Tesque) and 100 μg/ml streptomycin (Nacalai Tesque). BMDMs were differentiated in RPMI medium (Nacalai Tesque) including 10% FBS, 10% L929 cell (ATCC) supernatant, 100 U/ml penicillin and 0.1 mg/ml streptomycin for 6 days.

Before infection, macrophages were seeded into 6-, 24, 96 well-plates at a density of 2.5 × 10^6^, 3 × 10^5^, 1 × 10^5^ cells and pre-stimulated with IFN-γ (10 ng/ml) for 24 h to induce GBP2. For infection with *C. koseri*, the bacteria was pre-cultured with LB medium for 4 h under aerobic conditions at 37°C. The bacteria was subcultured (1:10) in fresh LB medium for 20 h to stationary phase. Bacterial density was calculated from the OD_600nm_ value, and the cells were resuspended into antibiotic-free medium at the indicated multiplicity of infection (MOI). BMDMs were infected with the bacteria (MOI = 10 or indicated MOI and centrifuged for 15 min at 500 × g and then BMDMs were incubated for 1 h at 37°C. After 1 h, 100 μg/ml gentamycin (Invitrogen) was added to kill extracellular bacteria. After 1 h incubation, the cells were washed once with PBS and changed fresh macrophage medium containing 10 μg/ml gentamicin for the remainder of the infection. The cells or supernatants were collected at 16 h or indicated times after infection.

### Transfection of LPS, Poly-dAdT or Flagellin and LPS Electroporation

BMDMs were pre-stimulated with 100 ng/ml Pam3CSK4 (Invivogen) for 4 h in macrophage medium. 2 μg/ml ultrapure LPS *E. coli* O111:B4 (Invivogen), 1 μg/ml poly-dAdT (Invivogen) or 10 μg/ml flagellin (invivogen) was transfected in serum-free RPMI medium including with 0.25% FuGeneHD (Promega) for 16 h or indicated times. Different from the standard protocol (33), we did not use Opti-MEM to avoid saturation of cell death in Atg12^Δmyeloid^ cells or *Gate-16*^−/−^*Gabarap*^−/−^ cells. BMDMs were pre-stimulated with Pam3CSK4 for 4 h and washed with PBS two times. The cells were electroporated with 30 μg/ml LPS using the 2D-Nucleofector system (Lonza) for 16 h.

### LPS and Cholera Toxin B Subunit (CTB) Treatment

BMDMs were pre-stimulated with 100 ng/ml Pam3CSK4 for 4 h. The cells were treated with non-treatment or 1 μg/ml LPS alone or 20 μg/ml CTB (List biological laboratories) or LPS and CTB treatment for indicated times.

### Cytokine and LDH Release Measurement

The concentrations of secreted mouse cytokines IL-1β, IL-6, IL-12 p40, TNF-α were measured by ELISA according to the manufacturer's protocol (eBioscience). IL-18 levels were tested by ELISA (MBL).

LDH release was measured by using CytoTox96 Non-Radio Cytotoxicity Assay kit (Promega). To calculate % of LDH release, values of [(sample–untreated sample)/(total cell lysate-untreated sample)] × 100 were calculated in accordance with the manufacture's instruction.

### Western Blot Analysis and Immunoprecipitation

The cells were lysed in a lysis buffer containing 1% Nonidet P-40, 150 mM NaCl, 20 mM Tris-HCl (pH 7.5), 1 mM EDTA and protease inhibitor cocktail (Nacalai Tesque). The cell lysates were separated by SDS–PAGE, transferred to polyvinyl difluoride membranes and subjected to immunoblotting using the indicated antibodies.

For caspase-1 and caspase-11 cleavage assay, Culture supernatant of BMDMs was added with 10% Trichloroacetic acid (TCA) and 10% acetone overnight at −20°C. The supernatants were centrifuged for 30 min at 15,000 rpm, 4°C and wash cold acetone two times. And then, Pellets were dried up and lysed in RIPA buffer. The lysates were detected by using Novex NuPAGE^®^ SDS-PAGE Gel system (Thermo).

For immunoprecipitation, cell lysates were pre-cleared with Protein G-Sepharose™ (Amersham Pharmacia Biotech) for 2 h and then incubated with Protein G-Sepharose™ containing 1.0 μg of the indicated antibodies for 12 h with rotation at 4°C. The immunoprecipitants were washed four times with lysis buffer, eluted by boiling with Laemmli sample buffer and subjected to immunoblot analysis using the indicated antibodies.

### ASC Oligomerization Assay

2.5 × 10^6^ BMDMs were seeded in 6 cm dish and pre-stimulated with 100 ng/ml Pam3CSK4 for 4 h and transfected with 2 μg/ml LPS for 6 h. The cells were collected and lysed with the 100 μl buffer A containing 20 mM HEPES-KOH (pH 7.5), 10 mM KCl, 1.5 mM MgCl_2_, 1 mM EDTA, 1 mM EGTA, and 320 mM sucrose. The lysates were centrifuged for 8 min at 300 × g, 4°C and the supernatants were mixed with the same volume of CHAPS buffer. The lysates were centrifuged for 8 min at 2,650 × g, 4°C and the supernatants were removed and the pellets were incubated with 20 μl CHAPS buffer containing 2 mM DSS (Thermo) for 2 h on ice. And then 3 × sample buffer was mixed with the lysates and boiled for 5 min, 98°C. ASC oligomerization was analyzed by western blot.

### Isolation and Treatment of Bacterial OMVs

OMVs were isolated from *E. coli* BL21 as described previously (44). Briefly, *E. coli* (BL21) were cultured overnight, and sub-cultured 1/1000 in 500 ml LB broth media and cultured 37°C overnight. The media were centrifuged at 10,000 × g for 10 min at 4°C, then the supernatant were filtered through 0.45 μm filter and 0.22 μm to remove whole bacteria and debris. The solutions were centrifuged at 150,000 × g for 3 h at 4°C to pellet OMVs. The pellets were re-suspended in sterile PBS. The concentration of OMVs was measured by protein assay and parts of OMVs solution were plated on the LB plate to confirm the bacterial free conditions. To stimulate BMDMs, BMDMs were seeded in the six well-plate for caspase-1 and caspase-11 cleavage assay and 96 well-plate for ELISA and LDH assay 1 day before stimulation. Isolated OMVs were treated with each 30 μg/well and 10 μg/ml in serum-free RPMI medium for 16 h.

### Immunofluorescence

BMDMs were seeded and cultured on the glass coverslips. The cells were pre-stimulated with 100 ng/ml Pam3CSK4 for 4 h and transfected with 5 μg/ml FITC-conjugated LPS (Sigma) or 2 μg/ml ultrapure LPS *E. coli* O111:B4 for 2 h. The cells were then fixed for 10 min in PBS containing 3.7% formaldehyde, permeabilized with PBS containing 0.1% Triton X-100 and blocked with 8% FBS in PBS. The cells were stained with rabbit anti-ASC antibody (1:100), rabbit anti-Gbp2 antibody (1:100), rabbit p62 antibody (1:100), mouse anti-HA antibody (1:400), mouse anti-ubiquitin (1:800), rat anti-caspase-11 (1:100) for 1 h at room temperature, followed by stained with Alexa-Fluor-488-conjugated or Alexa-Fluor-594-conjugated anti-mouse-IgG, Alexa-Fluor-594-conjugated anti-rat-IgG (Invitrogen), Alexa-Fluor-594-conjugated or Alexa-Fluor-647-conjugated anti-rabbit-IgG (Invitrogen) for 1 h at room temperature in the dark. For anti-ASC staining, fixed cells were blocked and permeabilized with Blocking Buffer (1 × PBS 5% normal serum/0.3% Trito X-100) for 1 h. Subsequently, cells were stained with anti-ASC (1:400) diluted in Antibody Dilution Buffer (1 × PBS/1% BSA/0.3% Triton X-100) for 12 h at 4°C and followed by the secondary antibody staining as described above. Nuclei were counterstained with DAPI (Nacalai Tesque). Finally, the immunostained cells were mounted with PermaFluor (Thermo Scientific) on glass slides and analyzed by confocal laser microscopy (FV1200 IX-83, Olympus) To quantify the number of GBP2-containing puncta in each cell, images of a single cell that was stained for GBP2 were analyzed by the ImageJ software (US National Institutes of Health).

### Quantification of Activated Caspase-1

Pam3CSK4 pre-stimulated BMDMs were transfected with LPS for 4 h and added with FAM-FLICA (ImmunoChemistly technology) at 3 h. The cells were washed with FACS buffer containing 2% FBS, 0.009% NaN_3_, 2 mM EDTA and PBS two times. Activated caspase-1 was detected by using flow cytometry using FACS Verse (Becton Dickinson) and quantified using FlowJo Software (Tree Star).

### Canonical Inflammasome Activation and Quantification of Damaged Mitochondria

To activate canonical inflammasome for the ELISA and WB, BMDMs were stimulated with 10 ng/ml LPS for 5 h and 5 mM ATP for 3 h. BMDMs were stimulated with 10 ng/ml LPS and 1 mM ATP for 30 min at 37°C. To stain the mitochondria, the cells were stained with 25 nM of MitoTracker Green FM and MitoTracker Deep Red FM (Invivogen) for 15 min at 37°C (67). The cells were washed with FACS buffer two times and the damaged mitochondria were analyzed by using flow cytometry.

### Isolation of Cytosol Fraction From BMDMs and LPS Quantification

To isolate the cytosol fraction from BMDMs, Digitonin-based fractionation method were utilized as described previously (44). 7.5 × 10^5^ BMDMs were seeded on the 24 well-plate and cultured overnight. The cells were transfected with 1 μg/well LPS for each time points and washed with cold PBS four times. Cells were treated with 180 μl of 0.005% digitonin extraction buffer for 8 min and the supernatant was collected for cytosol fraction. The non-cytosol fraction containing cell membrane, organelles and nucleus was collected in 180 μl of 0.1% CHAPS buffer. The quantity of LPS in each fraction was measured by using LAL Chromogenic Endotoxin Quantitation kit (Thermo).

### Reconstitution of BMDMs With Gate-16, Gabarap, GBP2 and These Mutants

Retroviral expression vectors of Gate-16, Gabarap and GBP2 were described as previously (42, 63). Retrovirus producing each Gate-16 and GBP2 were infected with 2 × 10^6^ *Gate-16*^−/−^*Gabarap*^−/−^ or *Gate-16*^−/−^*Gabarap*^−/−^*Gbp2*^−/−^ BMDMs. After 2 days, the cells were performed drug selection by addition of the 2 μg/ml blasticidin for 3 days.

### LPS Challenge *in vivo*

Mice were intraperitoneally injected with 10 mg/kg poly(I:C) (GE Healthcare) for 7 h and then 0.1 mg/kg or 0.4 mg/kg LPS were injected. After 3 h, the sera were collected from each mice and survival rates were tested. To prevent TLR4 signaling, 20 mg/kg of TAK-242 (CS-0408; Chemscene) was intraperitoneally injected at 0.5 h prior to LPS injection and at 0 h or 0.5 h post-LPS injection. To make the graph, the survival rate and cytokine concentration were used Prism5 software (Graph Pad software).

### Cecal Ligation Puncture (CLP)

The mice were anesthetized using pentobarbital by injecting intraperitoneally. Anesthetized mice were removed the hairs of the abdomen by shaver. The skin was disinfected with 75% Ethanol swab. The mouse made about 1 cm insection of the abdomen and took out the cecum from the cut line. Forty percent of the cecum was ligated and punctured once with a 21-gauge needle. Mice were monitored daily by the sign of a moribund state for lethality. For cytokine production, mice were sacrificed at 16 h post-CLP, then collected the peritoneal fluids and measured IL-1β and IL-6 by ELISA.

### Statistical Analysis

All experiments were performed using randomly assigned mice without investigator blinding. All data points and *n-*values reflect biological replicates (from three or four independent experiments). No data were excluded. Sample sizes were chosen by standard methods to ensure adequate power. All statistical analysis was performed using Excel 2013 (Microsoft) or GraphPad Prism5 (GraphPad Software). Statistical analyses for differences between two groups were performed using an unpaired two-tailed Student's *t-*test or log-rank test for analysis of the survival. *P* < 0.05 was considered to be statistically significant. No statistical methods were used to pre-determine sample size.

## Data Availability Statement

The raw data supporting the conclusions of this article will be made available by the authors, without undue reservation.

## Ethics Statement

The animal study was reviewed and approved by the Animal Research Committee of Research Institute for Microbial Diseases in Osaka University.

## Author Contributions

MY conceptualized and supervised this project. NS, MS, and HB performed experiments and analyzed the data. YL, AP, and JM prepared reagents, samples, and animals for this study. MY, NS, MS, and HB wrote this manuscript. All authors contributed to the article and approved the submitted version.

## Conflict of Interest

The authors declare that the research was conducted in the absence of any commercial or financial relationships that could be construed as a potential conflict of interest.

## Supplementary Material

Supplementary Figure 1 Normal activation of canonical NLRP3 inflammasome in *Gate-16*^−/−^*Gabarap*^−/−^ macrophages. **(A)** Release of IL-1β from wild-type (WT), *Atg12*^ΔMyeloid^, *Lc3a*^−/−^*, Lc3b*^−/−^, *Gate-16*^−/−^*, Gabarap*^−/−^*, Gabarapl1*^−/−^*, Lc3a*^−/−^*Lc3b*^−/−^, and *Gate-16*^−/−^*Gabarap*^−/−^ BMDMs primed with LPS followed by ATP treatment for 3 h. **(B)** Release of LDH from wild-type (WT), *Atg12*^ΔMyeloid^, *Lc3a*^−/−^*Lc3b*^−/−^, and *Gate-16*^−/−^*Gabarap*^−/−^ BMDMs primed with LPS followed by ATP treatment for 3 h. **(C)** Western blot analysis of cleaved caspase-1 (p20) in cell supernatants, and of pro-caspase-1 and Actin (loading control) in cell extracts of wild-type (WT) and *Gate-16*^−/−^*Gabarap*^−/−^ BMDMs primed with LPS followed by ATP treatment for 3 h. **(D,E)** Flow cytometry **(D)** and its quantification **(E)** of wild-type (WT), *Atg12*^ΔMyeloid^, *Lc3a*^−/−^*Lc3b*^−/−^, and *Gate-16*^−/−^*Gabarap*^−/−^ BMDMs primed with LPS followed by ATP stimulation for 3 h. Cells were stained for Mito Tracker Deep Red and Mito Tracker Green before ATP treatment. **(F)** Release of IL-1β from wild-type (WT), *Gate-16*^−/−^*Gabarap*^−/−^ and *Aim2*^−/−^ BMDMs primed with Pam3CSK4 followed by transfection of 1 μg/ml poly-dAdT for 16 h. **(G)** Release of IL-1β from wild-type (WT), *Gate-16*^−/−^*Gabarap*^−/−^ and *Nlrc4*^−/−^ BMDMs primed with Pam3CSK4 followed by transfection of 10 μg/ml flagellin for 16 h. **(H)** Release of IL-6 from wild-type (WT), *Atg12*^ΔMyeloid^, *Lc3a*^−/−^*, Lc3b*^−/−^, *Gate-16*^−/−^*, Gabarap*^−/−^*, Gabarapl1*^−/−^*, Lc3a*^−/−^*Lc3b*^−/−^, and *Gate-16*^−/−^*Gabarap*^−/−^ BMDMs primed with Pam3CSK4 followed by LPS transfection for 16 h. The data are representative of three independent experiments **(C,D)** and are combined data of more than three independent experiments **(A,B,E–H)**. ^*^*P* < 0.05, ^**^*P* < 0.01, ^***^*P* < 0.001, and N.S., not significant, two-tailed *t*-test.

Supplementary Figure 2 Increased release of caspase-11 in supernatants of *Gate-16*^−/−^*Gabarap*^−/−^ macrophages in response to OMV or *C. koseri* infection. **(A)** Western blot analysis of caspase-11 and cleaved caspase-1 (p20) released in cell supernatants, and of pro-caspase-1 and pro-caspase-11 and Actin (loading control) in cell extracts of wild-type (WT) and *Gate-16*^−/−^*Gabarap*^−/−^ BMDMs treated with OMV for 16 h. **(B)** Western blot analysis of caspase-11 and cleaved caspase-1 (p20) released in cell supernatants, and of pro-caspase-1 and pro-caspase-11 and Actin (loading control) in cell extracts of wild-type (WT) *Gate-16*^−/−^, *Gabarap*^−/−^ and *Gate-16*^−/−^*Gabarap*^−/−^ BMDMs pre-treated with IFN-γ for 24 h and subsequently infected with *C. koseri* for 16 h. **(C)** Western blot analysis of NLRP3, GBP2 and pro-IL-1β and Actin (loading control) in cell extracts of wild-type (WT) and *Gate-16*^−/−^*Gabarap*^−/−^ BMDMs unstimulated or stimulated with Pam3CSK4 alone. The data are representative of three independent experiments **(A–C)**.

Supplementary Figure 3 GBP2 but not TLR4 is involved in the caspase-11-dependent septic shock. **(A,B)** Poly(I:C) primed-wild type (WT: *n* = 18), *Gbp2*^−/−^ (*n* = 17) and *Casp11*^−/−^ (*n* = 13) mice were injected with 0.4 mg/kg LPS and collected serums at 3 h. After LPS injection, survival rate **(A)** and IL-1β in the sera **(B)** were investigated. **(C,D)** Naïve wild type mice untreated (*n* = 5) or treated with 20 mg/kg TAK-242 at 0.5 h prior to LPS injection, 0 or 0.5 h post-LPS infection (*n* = 10). The mice were injected with 30 mg/kg LPS. After LPS injection, survival rate **(C)** and indicated cytokines in the sera at 3 h **(D)** were investigated. **(E,F)** Poly(I:C)-primed *Gate-16*^−/−^*Gabarap*^−/−^ mice untreated (*n* = 5) or treated with 20 mg/kg TAK-242 at 0.5 h prior to LPS injection, 0 or 0.5 h post-LPS infection (*n* = 6). The mice were injected with 0.1 mg/kg LPS and collected serums at 3 h. After LPS injection, survival rate **(E)** and indicated cytokines in the sera **(F)** were investigated. The data are combined data of more than three independent experiments **(A–F)**. Log-rank test **(A,C,E)** and two-tailed Student *t*-test **(B,D,F)** ^*^*P* < 0.05, ^**^*P* < 0.01, and ^***^*P* < 0.001; N.S., not significant.

Supplementary Figure 4 GBP2 is required for the caspase-11 inflammasome activation but not for canonical inflammasome. **(A)** Western blot analysis of cleaved caspase-1 (p20) and caspase-11 in cell supernatants, and of pro-caspase-1 and pro-caspase-11 and Actin (loading control) in cell extracts of wild-type (WT), *Casp11*^−/−^ and *Gbp2*^−/−^ BMDMs primed with Pam3CSK4 followed by LPS transfection for 16 h. **(B)** Western blot analysis of cleaved caspase-1 (p20) and caspase-11 in cell supernatants, and of pro-caspase-1 and pro-caspase-11 and Actin (loading control) in cell extracts of wild-type (WT), *Casp11*^−/−^ and *Gbp2*^−/−^ BMDMs treated with OMV for 16 h. **(C,D)** Release of IL-1β **(C)** or LDH **(D)** from wild-type (WT), *Gbp2*^−/−^ and *Casp11*^−/−^BMDMs treated with OMV for 16 h. **(E,F)** Release of IL-1β **(E)** or LDH **(F)** from wild-type (WT) and *Gbp2*^−/−^ BMDMs primed with LPS followed by ATP treatment for 3 h. **(G)** Western blot analysis of cleaved caspase-1 (p20) in cell supernatants, and of pro-caspase-1 and Actin (loading control) in cell extracts of wild-type (WT), *Gbp2*^−/−^ and *Casp1*^−/−^ BMDMs primed with LPS followed by ATP treatment for 3 h. **(H)** LAL assay for LPS (EU, endotoxin units) in the cytosolic and residual non-cytosolic fractions of wild-type (WT) and *Gate-16*^−/−^*Gabarap*^−/−^ BMDMs non-transfected or transfected with LPS at indicated concentrations for 2 h obtained by digitonin fractionation. The data are representative of three independent experiments **(A,B,G)** and are combined data of three independent experiments **(C–F,H)**. ^***^*P* < 0.001; N.S., not significant, two-tailed Student *t*-test.

Supplementary Figure 5 Reintroduction of Gabarap in DKO cells partially restores IL-1β production. **(A,B)** Quantification of GBP2 puncta. The numbers **(A)** and total areas **(B)** of GBP2 puncta in wild-type (WT) and Gate-16^−/−^*Gabarap*^−/−^ BMDMs stimulated by LPS transfection for 6 h and immunostained for GBP2 were quantified by using ImageJ software. **(C,D)** Quantification of GBP2 puncta. The numbers **(C)** and total areas **(D)** of GBP2 puncta in Gate-16^−/−^*Gabarap*^−/−^ BMDMs stably expressing HA-tagged wild-type Gate-16 (WT Gate-16), the G116A mutant (G116A Gate-16) and empty vectors. The cells were stimulated by LPS transfection for 6 h and immunostained for GBP2 were quantified by using ImageJ software. **(E)** Release of IL-1β from Gate-16^−/−^*Gabarap*^−/−^ BMDMs stably expressing Gate-16, Gabarap or empty vector. The cells were primed with Pam3CSK4 and subsequently stimulated by LPS transfection for 16 h. **(F)** Release of IL-1β from wild-type (WT), *Gate-16*^−/−^*Gabarap*^−/−^ and *Gate-16*^−/−^*Gabarap*^−/−^*Gbp2*^−/−^ BMDMs primed with Pam3CSK4 followed by LPS transfection for indicated hours. **(G)** Release of LDH from wild-type (WT), *Gate-16*^−/−^*Gabarap*^−/−^ and *Gate-16*^−/−^*Gabarap*^−/−^*Gbp2*^−/−^ BMDMs primed with Pam3CSK4 followed by LPS transfection for 16 h. **(H)** Release of IL-1β from wild-type (WT), *Gate-16*^−/−^*Gabarap*^−/−^ and *Gate-16*^−/−^*Gabarap*^−/−^*Gbp2*^−/−^ BMDMs pre-treated with IFN-γ for 24 h and subsequently infected with *C. koseri* for indicated hours. **(I)** Release of IL-1β from wild-type (WT) and *Gbp2*^−/−^ BMDMs pre-treated with IFN-γ for 24 h and subsequently infected with *C. koseri* for indicated hours. The data re combined data of more than three independent experiments **(A–I)**. ^*^*P* < 0.05, ^**^*P* < 0.01, and ^***^*P* < 0.001, two-tailed *t*-test.

Supplementary Figure 6 Generation of *Atg12*^fl/fl^, *Casp1*^−/−^, *Casp11*^−/−^, *Aim2*^−/−^ or *Nlrc4*^−/−^ mice. **(A–D)** Schematic of the gRNA-targeting sites in the *Casp1* **(A)** and *Casp11* **(B)**, *Aim2* **(C)**, and *Nlrc4* **(D)** genes. Boxed and underlined regions are the gRNA target or PAM sequences, respectively. Green letters indicate insertion. **(E)** Schematic of the gRNA-targeting sites in the *Atg12* gene, targeting fragments containing the exon flanked by loxP sequences. **(F)** Western blot analysis of pro-caspase-1 and pro-caspase-11 and Actin (loading control) in cell extracts of wild-type (WT), *Casp1*^−/−^ and *Casp11*^−/−^ BMDMs primed with LPS for 16 h. **(G,H)** Western blot analysis of indicated proteins in cell extracts of wild-type (WT), *Aim2*^−/−^ **(G)** or *Nlrc4*^−/−^ **(H)** BMDMs primed with LPS for 16 h. **(I)** Western blot analysis of Atg12 and Actin (loading control) in cell extracts of *Atg12*^fl/fl^ (control) or LysM-Cre *Atg12*^fl/fl^ (*Atg12*^Δmyeolid^) BMDMs. Note that Atg12 is conjugated with Atg5, therefore, the molecular weight is higher than expected. The data are representative of three independent experiments **(F–I)**.
