# Supplementary figures and images for: Role of Gate-16 and Gabarap in Prevention of Caspase-11-Dependent Excess Inflammation and Lethal Endotoxic Shock
Source: Front Immunol. 2020 Sep 15;11:561948. doi: 10.3389/fimmu.2020.561948 (PMC7522336; doi:10.3389/fimmu.2020.561948)

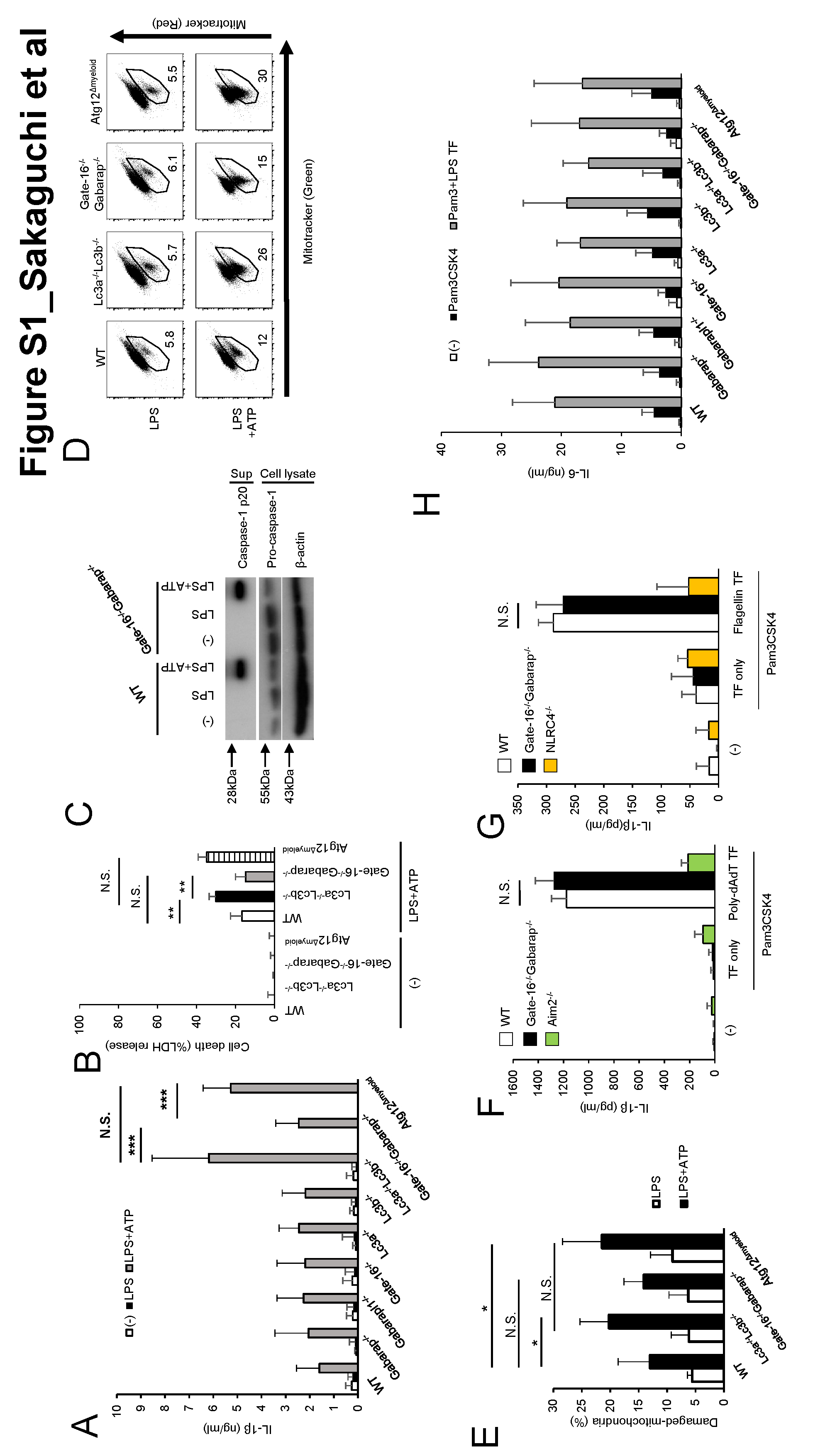

Supplement: Supplementary Figure 1 — Normal activation of canonical NLRP3 inflammasome in Gate-16−/−Gabarap−/− macrophages. (A) Release of IL-1β from wild-type (WT), Atg12ΔMyeloid, Lc3a−/−, Lc3b−/−, Gate-16−/−, Gabarap−/−, Gabarapl1−/−, Lc3a−/−Lc3b−/−, and Gate-16−/−Gabarap−/− BMDMs primed with LPS followed by ATP treatment for 3 h. (B) Release of LDH from wild-type (WT), Atg12ΔMyeloid, Lc3a−/−Lc3b−/−, and Gate-16−/−Gabarap−/− BMDMs primed with LPS followed by ATP treatment for 3 h. (C) Western blot analysis of cleaved caspase-1 (p20) in cell supernatants, and of pro-caspase-1 and Actin (loading control) in cell extracts of wild-type (WT) and Gate-16−/−Gabarap−/− BMDMs primed with LPS followed by ATP treatment for 3 h. (D,E) Flow cytometry (D) and its quantification (E) of wild-type (WT), Atg12ΔMyeloid, Lc3a−/−Lc3b−/−, and Gate-16−/−Gabarap−/− BMDMs primed with LPS followed by ATP stimulation for 3 h. Cells were stained for Mito Tracker Deep Red and Mito Tracker Green before ATP treatment. (F) Release of IL-1β from wild-type (WT), Gate-16−/−Gabarap−/− and Aim2−/− BMDMs primed with Pam3CSK4 followed by transfection of 1 μg/ml poly-dAdT for 16 h. (G) Release of IL-1β from wild-type (WT), Gate-16−/−Gabarap−/− and Nlrc4−/− BMDMs primed with Pam3CSK4 followed by transfection of 10 μg/ml flagellin for 16 h. (H) Release of IL-6 from wild-type (WT), Atg12ΔMyeloid, Lc3a−/−, Lc3b−/−, Gate-16−/−, Gabarap−/−, Gabarapl1−/−, Lc3a−/−Lc3b−/−, and Gate-16−/−Gabarap−/− BMDMs primed with Pam3CSK4 followed by LPS transfection for 16 h. The data are representative of three independent experiments (C,D) and are combined data of more than three independent experiments (A,B,E–H). *P < 0.05, **P < 0.01, ***P < 0.001, and N.S., not significant, two-tailed t-test. [file Image_1.JPEG]

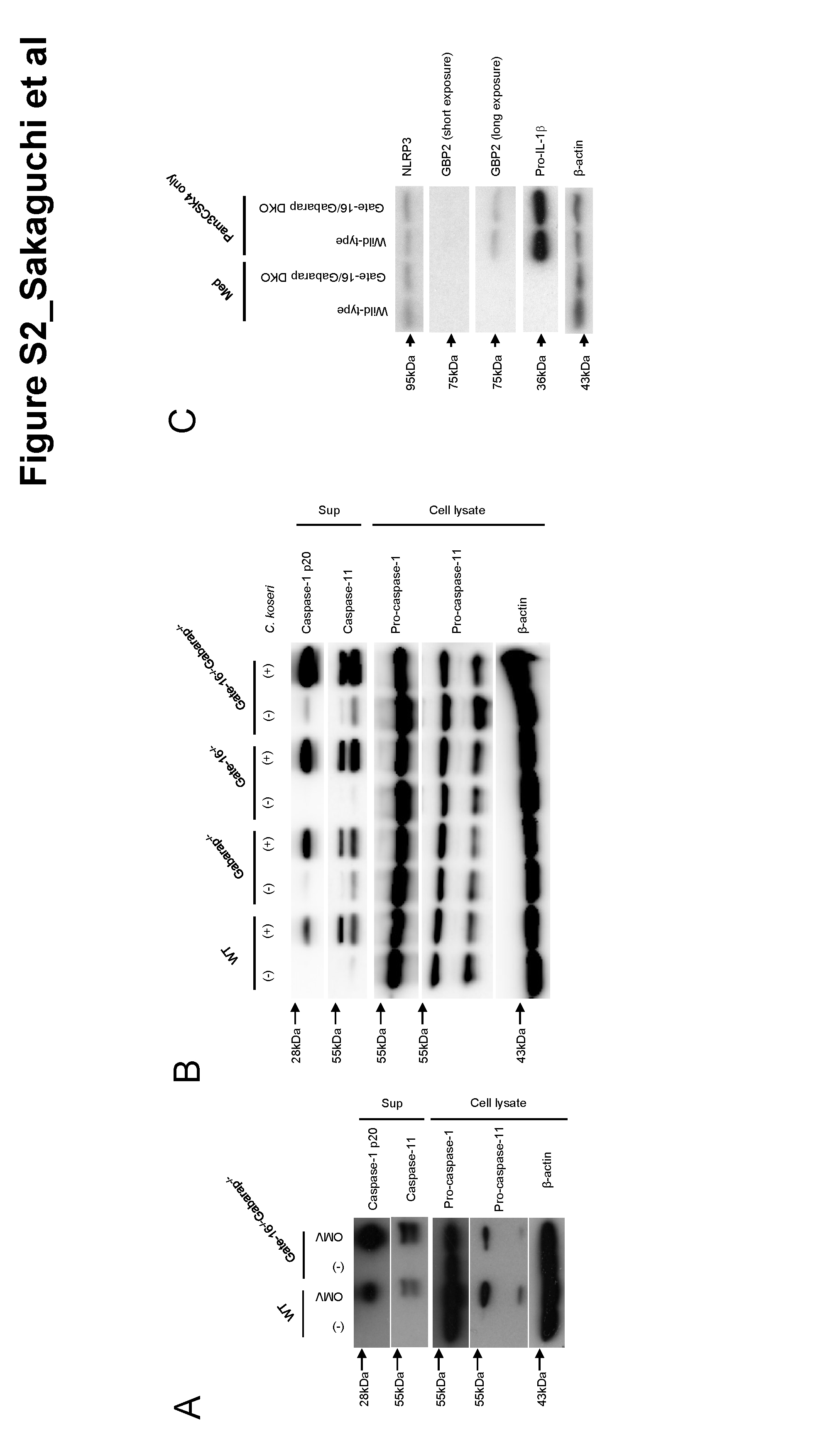

Supplement: Supplementary Figure 2 — Increased release of caspase-11 in supernatants of Gate-16−/−Gabarap−/− macrophages in response to OMV or C. koseri infection. (A) Western blot analysis of caspase-11 and cleaved caspase-1 (p20) released in cell supernatants, and of pro-caspase-1 and pro-caspase-11 and Actin (loading control) in cell extracts of wild-type (WT) and Gate-16−/−Gabarap−/− BMDMs treated with OMV for 16 h. (B) Western blot analysis of caspase-11 and cleaved caspase-1 (p20) released in cell supernatants, and of pro-caspase-1 and pro-caspase-11 and Actin (loading control) in cell extracts of wild-type (WT) Gate-16−/−, Gabarap−/− and Gate-16−/−Gabarap−/− BMDMs pre-treated with IFN-γ for 24 h and subsequently infected with C. koseri for 16 h. (C) Western blot analysis of NLRP3, GBP2 and pro-IL-1β and Actin (loading control) in cell extracts of wild-type (WT) and Gate-16−/−Gabarap−/− BMDMs unstimulated or stimulated with Pam3CSK4 alone. The data are representative of three independent experiments (A–C). [file Image_2.JPEG]

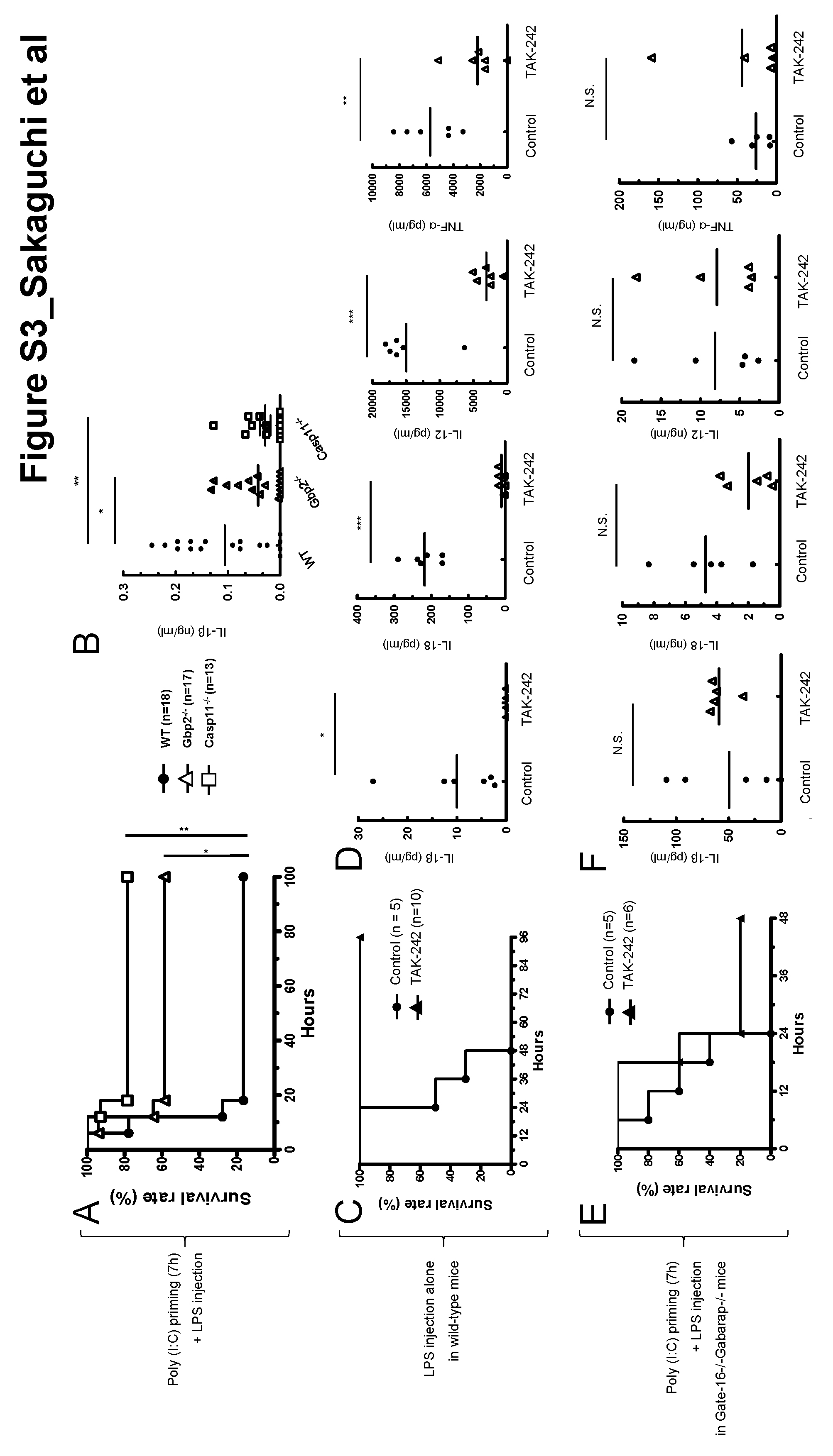

Supplement: Supplementary Figure 3 — GBP2 but not TLR4 is involved in the caspase-11-dependent septic shock. (A,B) Poly(I:C) primed-wild type (WT: n = 18), Gbp2−/− (n = 17) and Casp11−/− (n = 13) mice were injected with 0.4 mg/kg LPS and collected serums at 3 h. After LPS injection, survival rate (A) and IL-1β in the sera (B) were investigated. (C,D) Naïve wild type mice untreated (n = 5) or treated with 20 mg/kg TAK-242 at 0.5 h prior to LPS injection, 0 or 0.5 h post-LPS infection (n = 10). The mice were injected with 30 mg/kg LPS. After LPS injection, survival rate (C) and indicated cytokines in the sera at 3 h (D) were investigated. (E,F) Poly(I:C)-primed Gate-16−/−Gabarap−/− mice untreated (n = 5) or treated with 20 mg/kg TAK-242 at 0.5 h prior to LPS injection, 0 or 0.5 h post-LPS infection (n = 6). The mice were injected with 0.1 mg/kg LPS and collected serums at 3 h. After LPS injection, survival rate (E) and indicated cytokines in the sera (F) were investigated. The data are combined data of more than three independent experiments (A–F). Log-rank test (A,C,E) and two-tailed Student t-test (B,D,F) *P < 0.05, **P < 0.01, and ***P < 0.001; N.S., not significant. [file Image_3.JPEG]

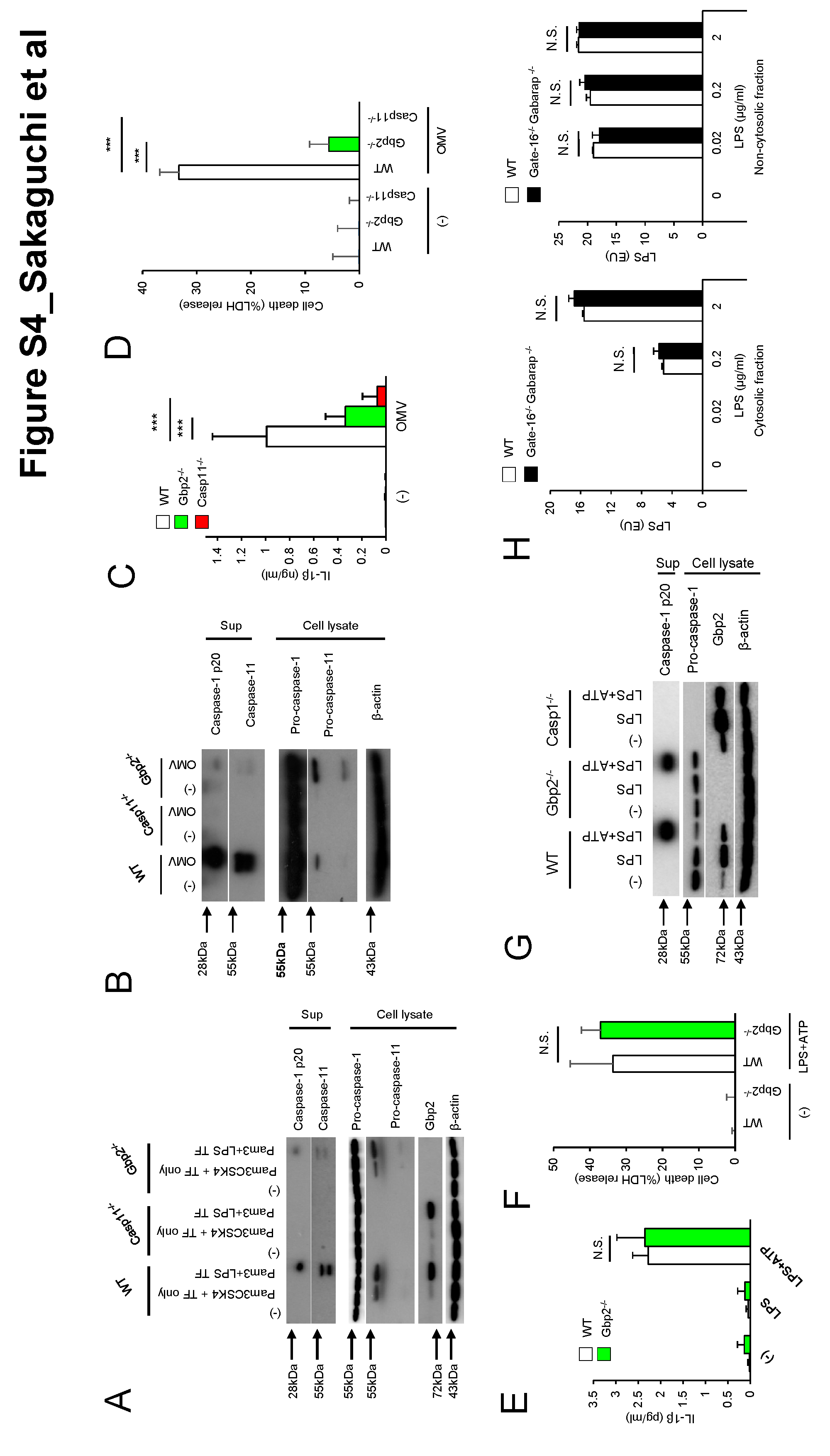

Supplement: Supplementary Figure 4 — GBP2 is required for the caspase-11 inflammasome activation but not for canonical inflammasome. (A) Western blot analysis of cleaved caspase-1 (p20) and caspase-11 in cell supernatants, and of pro-caspase-1 and pro-caspase-11 and Actin (loading control) in cell extracts of wild-type (WT), Casp11−/− and Gbp2−/− BMDMs primed with Pam3CSK4 followed by LPS transfection for 16 h. (B) Western blot analysis of cleaved caspase-1 (p20) and caspase-11 in cell supernatants, and of pro-caspase-1 and pro-caspase-11 and Actin (loading control) in cell extracts of wild-type (WT), Casp11−/− and Gbp2−/− BMDMs treated with OMV for 16 h. (C,D) Release of IL-1β (C) or LDH (D) from wild-type (WT), Gbp2−/− and Casp11−/−BMDMs treated with OMV for 16 h. (E,F) Release of IL-1β (E) or LDH (F) from wild-type (WT) and Gbp2−/− BMDMs primed with LPS followed by ATP treatment for 3 h. (G) Western blot analysis of cleaved caspase-1 (p20) in cell supernatants, and of pro-caspase-1 and Actin (loading control) in cell extracts of wild-type (WT), Gbp2−/− and Casp1−/− BMDMs primed with LPS followed by ATP treatment for 3 h. (H) LAL assay for LPS (EU, endotoxin units) in the cytosolic and residual non-cytosolic fractions of wild-type (WT) and Gate-16−/−Gabarap−/− BMDMs non-transfected or transfected with LPS at indicated concentrations for 2 h obtained by digitonin fractionation. The data are representative of three independent experiments (A,B,G) and are combined data of three independent experiments (C–F,H). ***P < 0.001; N.S., not significant, two-tailed Student t-test. [file Image_4.jpg]

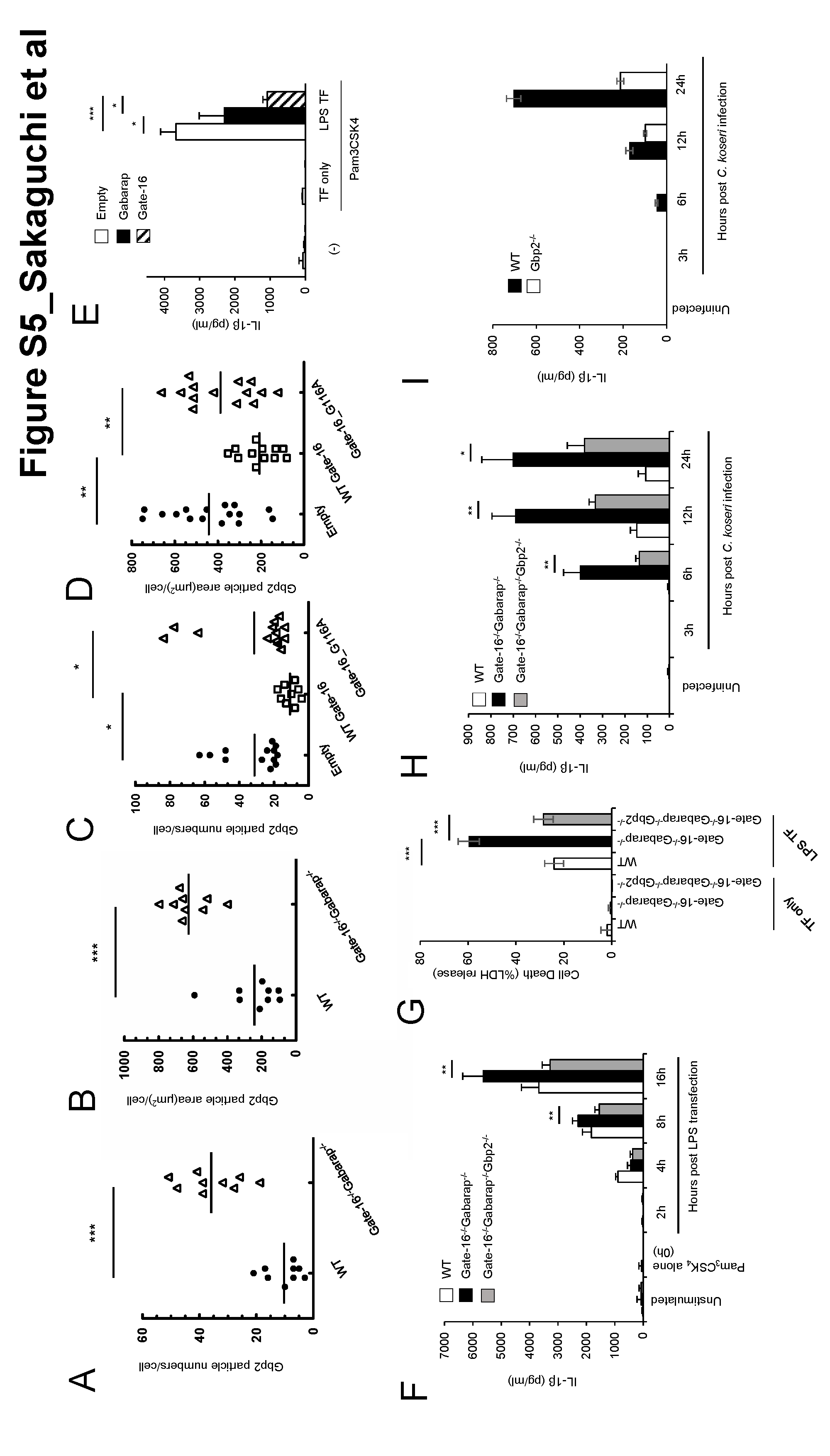

Supplement: Supplementary Figure 5 — Reintroduction of Gabarap in DKO cells partially restores IL-1β production. (A,B) Quantification of GBP2 puncta. The numbers (A) and total areas (B) of GBP2 puncta in wild-type (WT) and Gate-16−/−Gabarap−/− BMDMs stimulated by LPS transfection for 6 h and immunostained for GBP2 were quantified by using ImageJ software. (C,D) Quantification of GBP2 puncta. The numbers (C) and total areas (D) of GBP2 puncta in Gate-16−/−Gabarap−/− BMDMs stably expressing HA-tagged wild-type Gate-16 (WT Gate-16), the G116A mutant (G116A Gate-16) and empty vectors. The cells were stimulated by LPS transfection for 6 h and immunostained for GBP2 were quantified by using ImageJ software. (E) Release of IL-1β from Gate-16−/−Gabarap−/− BMDMs stably expressing Gate-16, Gabarap or empty vector. The cells were primed with Pam3CSK4 and subsequently stimulated by LPS transfection for 16 h. (F) Release of IL-1β from wild-type (WT), Gate-16−/−Gabarap−/− and Gate-16−/−Gabarap−/−Gbp2−/− BMDMs primed with Pam3CSK4 followed by LPS transfection for indicated hours. (G) Release of LDH from wild-type (WT), Gate-16−/−Gabarap−/− and Gate-16−/−Gabarap−/−Gbp2−/− BMDMs primed with Pam3CSK4 followed by LPS transfection for 16 h. (H) Release of IL-1β from wild-type (WT), Gate-16−/−Gabarap−/− and Gate-16−/−Gabarap−/−Gbp2−/− BMDMs pre-treated with IFN-γ for 24 h and subsequently infected with C. koseri for indicated hours. (I) Release of IL-1β from wild-type (WT) and Gbp2−/− BMDMs pre-treated with IFN-γ for 24 h and subsequently infected with C. koseri for indicated hours. The data re combined data of more than three independent experiments (A–I). *P < 0.05, **P < 0.01, and ***P < 0.001, two-tailed t-test. [file Image_5.jpg]

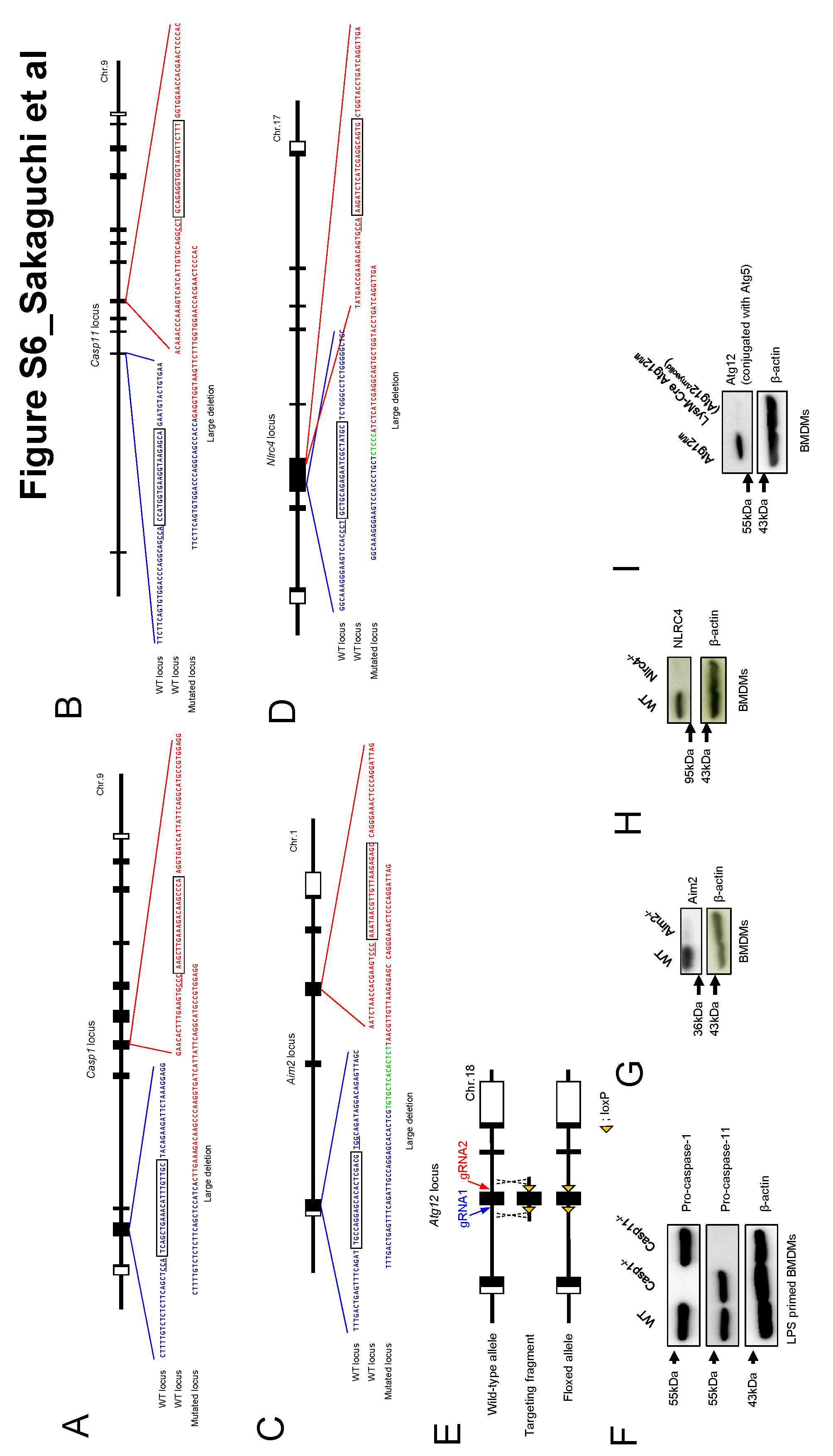

Supplement: Supplementary Figure 6 — Generation of Atg12fl/fl, Casp1−/−, Casp11−/−, Aim2−/− or Nlrc4−/− mice. (A–D) Schematic of the gRNA-targeting sites in the Casp1 (A) and Casp11 (B), Aim2 (C), and Nlrc4 (D) genes. Boxed and underlined regions are the gRNA target or PAM sequences, respectively. Green letters indicate insertion. (E) Schematic of the gRNA-targeting sites in the Atg12 gene, targeting fragments containing the exon flanked by loxP sequences. (F) Western blot analysis of pro-caspase-1 and pro-caspase-11 and Actin (loading control) in cell extracts of wild-type (WT), Casp1−/− and Casp11−/− BMDMs primed with LPS for 16 h. (G,H) Western blot analysis of indicated proteins in cell extracts of wild-type (WT), Aim2−/− (G) or Nlrc4−/− (H) BMDMs primed with LPS for 16 h. (I) Western blot analysis of Atg12 and Actin (loading control) in cell extracts of Atg12fl/fl (control) or LysM-Cre Atg12fl/fl (Atg12Δmyeolid) BMDMs. Note that Atg12 is conjugated with Atg5, therefore, the molecular weight is higher than expected. The data are representative of three independent experiments (F–I). [file Image_6.JPEG]

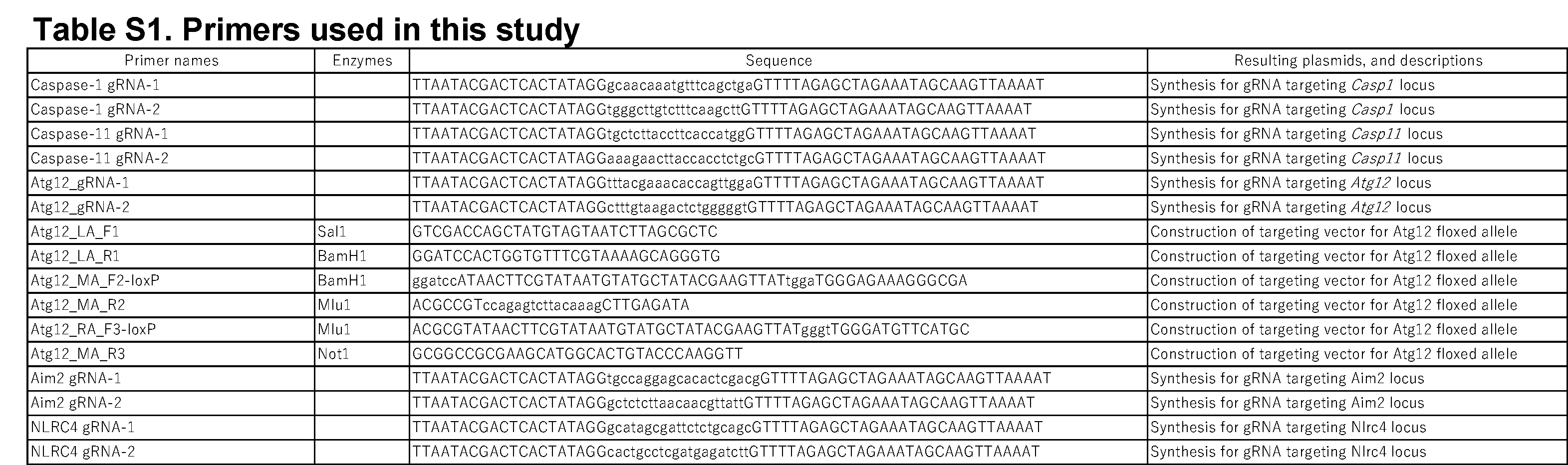

Supplement: Supplementary file 7 [file Image_7.JPEG]
